# DNA methylation mediates the effect of maternal smoking on offspring birthweight: a birth cohort study of multi-ethnic US mother–newborn pairs

**DOI:** 10.1186/s13148-021-01032-6

**Published:** 2021-03-04

**Authors:** Richard Xu, Xiumei Hong, Boyang Zhang, Wanyu Huang, Wenpin Hou, Guoying Wang, Xiaobin Wang, Tak Igusa, Liming Liang, Hongkai Ji

**Affiliations:** 1 Department of Computer Science, Whiting School of Engineering, Johns Hopkins University, Baltimore, MD USA; 2 Center On the Early Life Origins of Disease, Department of Population, Family and Reproductive Health, Johns Hopkins University Bloomberg School of Public Health, Baltimore, MD USA; 3 Department of Biostatistics, Johns Hopkins University Bloomberg School of Public Health, Baltimore, MD 21205 USA; 4 Department of Civil and Systems Engineering, Whiting School of Engineering, Johns Hopkins University, Baltimore, MD USA; 5 Department of Pediatrics, Johns Hopkins University School of Medicine, Baltimore, MD 21205 USA; 6 Department of Epidemiology, T.H. Chan School of Public Health, Harvard University, Boston, MA USA; 7 Department of Biostatistics, T.H. Chan School of Public Health, Harvard University, Boston, MA USA

**Keywords:** Maternal smoking, Low birthweight, DNA methylation, Epigenome-wide association study, Mediation analysis

## Abstract

**Background:**

Maternal smoking affects more than half a million pregnancies each year in the US and is known to result in fetal growth restriction as measured by lower birthweight and its associated long-term consequences. Maternal smoking also has been linked to altered fetal DNA methylation (DNAm). However, what remains largely unexplored is whether these DNAm alterations are merely markers of smoking exposure or if they also have implications for health outcomes. This study tested the hypothesis that fetal DNAm mediates the effect of maternal smoking on newborn birthweight.

**Methods:**

This study included mother–newborn pairs from a US predominantly urban, low-income multi-ethnic birth cohort. DNAm in cord blood were determined using the Illumina Infinium MethylationEPIC BeadChip. After standard quality control and normalization procedures, an epigenome-wide association study (EWAS) of maternal smoking was performed using linear regression models, controlling for maternal age, education, race, parity, pre-pregnancy body mass index, alcohol consumption, gestational age, maternal pregestational/gestational diabetes, child sex, cord blood cell compositions and batch effects. To quantify the degree to which cord DNAm mediates the smoking-birthweight association, the VanderWeele-Vansteelandt approach for single mediator and structural equational model for multiple mediators were used, adjusting for pertinent covariates.

**Results:**

The study included 954 mother–newborn pairs. Among mothers, 165 (17.3%) ever smoked before or during pregnancy. Newborns with smoking exposure had on average 258 g lower birthweight than newborns without exposure (*P* < 0.001). Using a false discovery rate (FDR) < 0.05 as the significance cutoff, the EWAS identified 38 differentially methylated CpG sites associated with maternal smoking. Of those, 17 CpG sites were mapped to previously reported genes: *GFI1, AHRR, CYP1A1, and CNTNAP2;* 8 of those, located in the first three genes, were Bonferroni significantly associated with newborn birthweight and mediated the smoking-birthweight association. The combined mediation effect of the three genes explained 67.8% of the smoking-birthweight association.

**Conclusions:**

Our study not only lends further support that maternal smoking alters fetal DNAm in a multiethnic population, but also suggests that fetal DNAm substantially mediates the maternal smoking–birthweight association. Our findings, if further validated, indicate that DNAm modification is likely an important pathway by which maternal smoking impairs fetal growth and, perhaps, even long-term health outcomes.

## Background

In the US, about 12% of pregnant women continue to smoke during pregnancy [1], which translates to more than half a million smoking-affected pregnancies each year [2]. Maternal smoking during pregnancy has been known to adversely affect fetal growth, as reflected in lower birthweight compared to the non-smoking group [3]. More recently, maternal smoking also has been linked to altered fetal DNA methylation (DNAm); these findings are consistent based on either self-reported smoking or objective measurement of blood cotinine levels [4–6]. The importance of these observations reaches far beyond the perinatal period. Low birthweight has been linked to a range of pediatric disorders [7] and chronic diseases later in life, including obesity, diabetes, cardiovascular diseases, and stroke, as highlighted by the Barker Hypothesis [8]. Such a link between low birthweight and chronic diseases was later confirmed by numerous studies around the world [9]. In recent years, prospective birth cohort studies [10, 11] have lent further support for the concept of developmental origins of health and disease (DoHaD) [12, 13].

Despite mounting evidence of DoHaD, the underlying biological mechanisms driving them remain to be understood. One plausible hypothesis points to epigenetic modification, which regulates gene expression without changing the original DNA sequence. Specifically, the epigenome operates at the interface between the genome and the environment; is largely established *in-utero;* and is particularly sensitive to early life environmental perturbation [14]. DNAm is a well-studied epigenetic mechanism, and its stability over decades makes it a very useful biomarker to study early life environmental exposures and life-course health [14]. DNAm in cord blood at multiple sites have also been found to be significantly associated with birthweight, as supported by an increasing number of studies [15–18] including the meta-*analysis in* the Pregnancy And Childhood Epigenetics Consortium [16]. Some of birthweight-related CpG sites overlap with those reported to be associated with maternal smoking [16]. In this context, maternal smoking, fetal DNAm and birthweight assessed together could serve as an exemplifying model to gain deeper insight into DoHaD.

This study aimed to fill in research gaps and to advance the field in the following ways. First, there is strong evidence that maternal smoking can affect fetal DNAm as measured in cord blood, and a list of related genes are consistently found across studies [4, 5, 19]. To date, all the related published studies were based on use of the Illumina HumanMethylation27 or 450 BeadChip, while a higher density BeadChip, with higher coverage of methylation dynamic regions, such as the enhancers, is now available. More importantly, a significant gap is the lingering lack of data for disadvantaged populations, especially US urban, low-income African American populations, who are at high-risk of low birthweight and chronic diseases. Second, despite robust findings for the association between maternal smoking and fetal DNAm, it remains to be determined whether the smoking-associated DNAm alterations *merely* represent markers of smoking exposure or also are biomarkers of smoking-associated adverse health effects. Mediation analyses could help to address this question by linking maternal smoking (exposure), DNAm (mediator) and birthweight (health outcome) together and dissecting the direct effects of smoking and indirect (mediation) effects of smoking via DNAm. To our knowledge, a limited number of studies of this kind were conducted in European samples using the 450 BeadChip, [20, 21] while none have been conducted with African Americans.

This study was designed to analyze data gathered from mother–newborn pairs enrolled in the Boston Birth Cohort, a predominantly urban, low-income multiethnic (Blacks and Hispanics) sample in Boston, MA. It sought to accomplish the following objectives: first, to conduct an epigenome-wide association study (EWAS) to identify novel genes and replicate known genes with altered methylation levels associated with maternal smoking during pregnancy, using the latest Illumina Infinium MethylationEPIC BeadChip. Second, to examine whether smoking-associated DNAm alterations are also associated with newborn birthweight. Third, to estimate to what degree smoking-associated methylation alterations in cord blood individually and collectively mediate the smoking-birthweight association. To our knowledge, this is the first large study of this kind in a high-risk US multiethnic population, with application of advanced mediation analyses to quantify individual CpG sites and their combined mediation of the smoking-birthweight association.

## Results

### Population characteristics

After data quality control steps (see “Methods” section), the current study included a total of 954 mother–newborn dyads; of those, 165 (17.3%) newborns were exposed to maternal ever smoking during pregnancy and 789 were unexposed. Compared with unexposed newborns, exposed newborns had 258 g lower birthweight (2911 vs. 3169 g, *P* < 0.001, Table 1). Compared to non-smokers, smoking mothers had higher pre-pregnancy BMI (*P* = 0.007), higher rates of alcohol consumption (*P* < 0.001), and lower education level (*P* < 0.001). Gestational age was slightly shorter in exposed newborns compared to non-exposed (38.3 vs. 38.7 weeks, *P* = 0.058). Other factors including maternal age at delivery, parity, maternal pregestational/gestational diabetes and newborn sex were comparable between the two groups (all *P* > 0.05, Table 1).

**Table 1:** Characteristics of 954 mother–newborn pairs from the Boston Birth Cohort by maternal smoking status during pregnancy

	No smoking	Smoking	*P*^a^
*N*	789	165	
Continuous variables [mean (SD)]			
Maternal age at delivery (years)	28.4 (6.6)	27.9 (6.1)	0.329
Maternal pre-pregnancy BMI (kg/m^2^)	26.7 (6.2)	28.1 (7.0)	0.007
Gestational age at delivery (weeks)	38.7 (2.4)	38.3 (2.7)	0.058
Child birthweight (g)	3169.1 (656.1)	2910.5 (680.6)	< 0.001
Categorical variables, *n* (%)			
Parity (≥ 1 live birth)	437 (55.4)	92 (55.8)	0.999
Maternal alcohol consumption	45 (5.7)	34 (20.6)	< 0.001
Maternal education level (> high school)	286 (36.2)	33 (20.0)	< 0.001
Maternal race			0.457
Black	566 (71.7)	113 (68.5)	
Non-Black	223 (28.3)	52 (31.5)	
Maternal diabetes			0.333
No	700 (88.7)	141 (85.5)	
Gestational diabetes	51 (6.5)	16 (9.7)	
Pregestational diabetes	38 (4.8)	8 (4.8)	
Child sex (male)	410 (52.0)	94 (57.0)	0.278

*SD* standard deviation, *BMI* body mass index

^a^Population characteristics was compared between those mothers who ever smoked during pregnancy versus those who did not, using the chi-square test for categorical variables and ANOVA for continuous variables

### EWAS with maternal smoking in cord blood

**Fig. 1:**
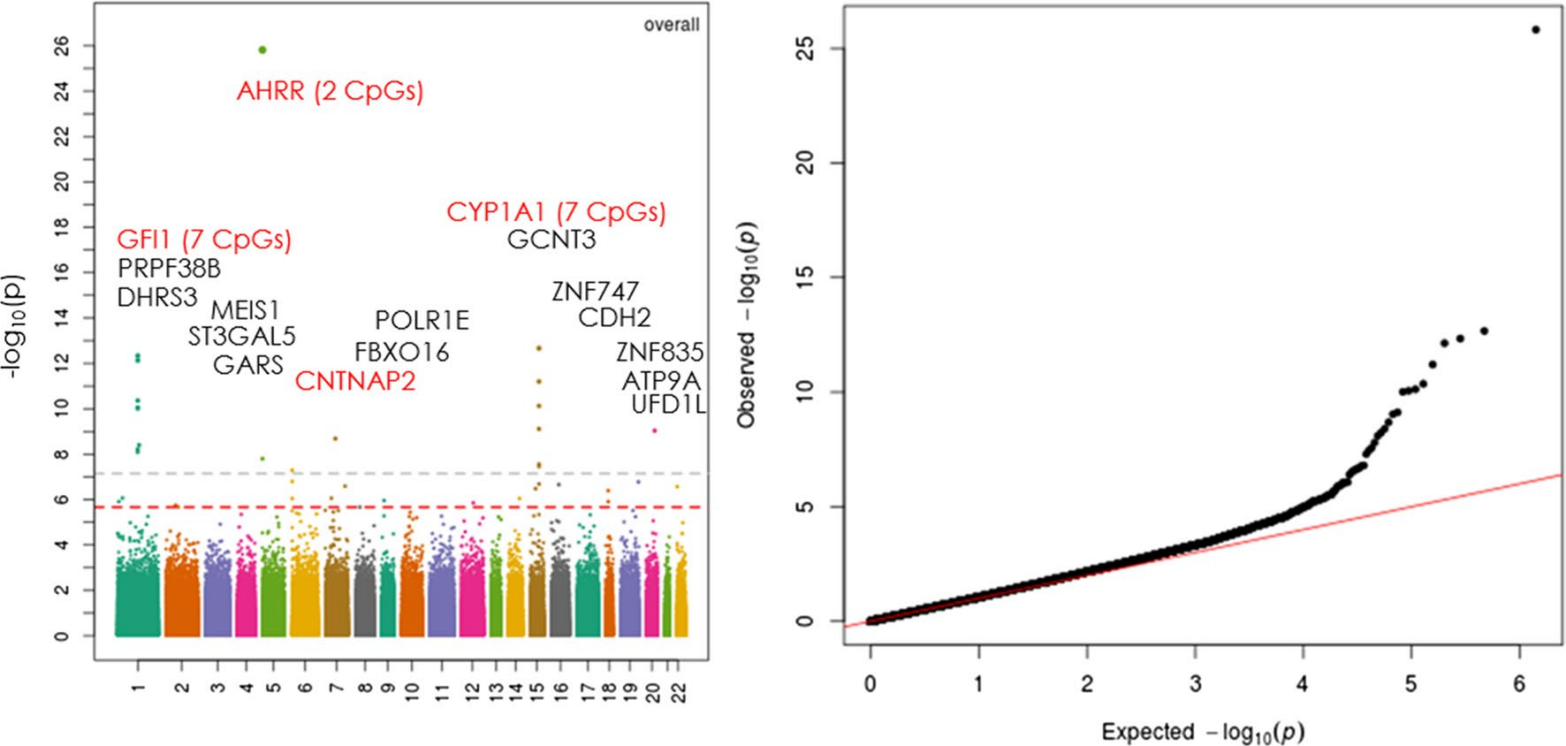
Manhattan plot of the association between maternal smoking during pregnancy and cord blood DNA methylation (left panel); and Quantile–Quantile plot of the association between maternal smoking during pregnancy and cord blood DNA methylation (right panel), in 954 mother–newborn pairs in the Boston Birth Cohort

**Fig. 2:**
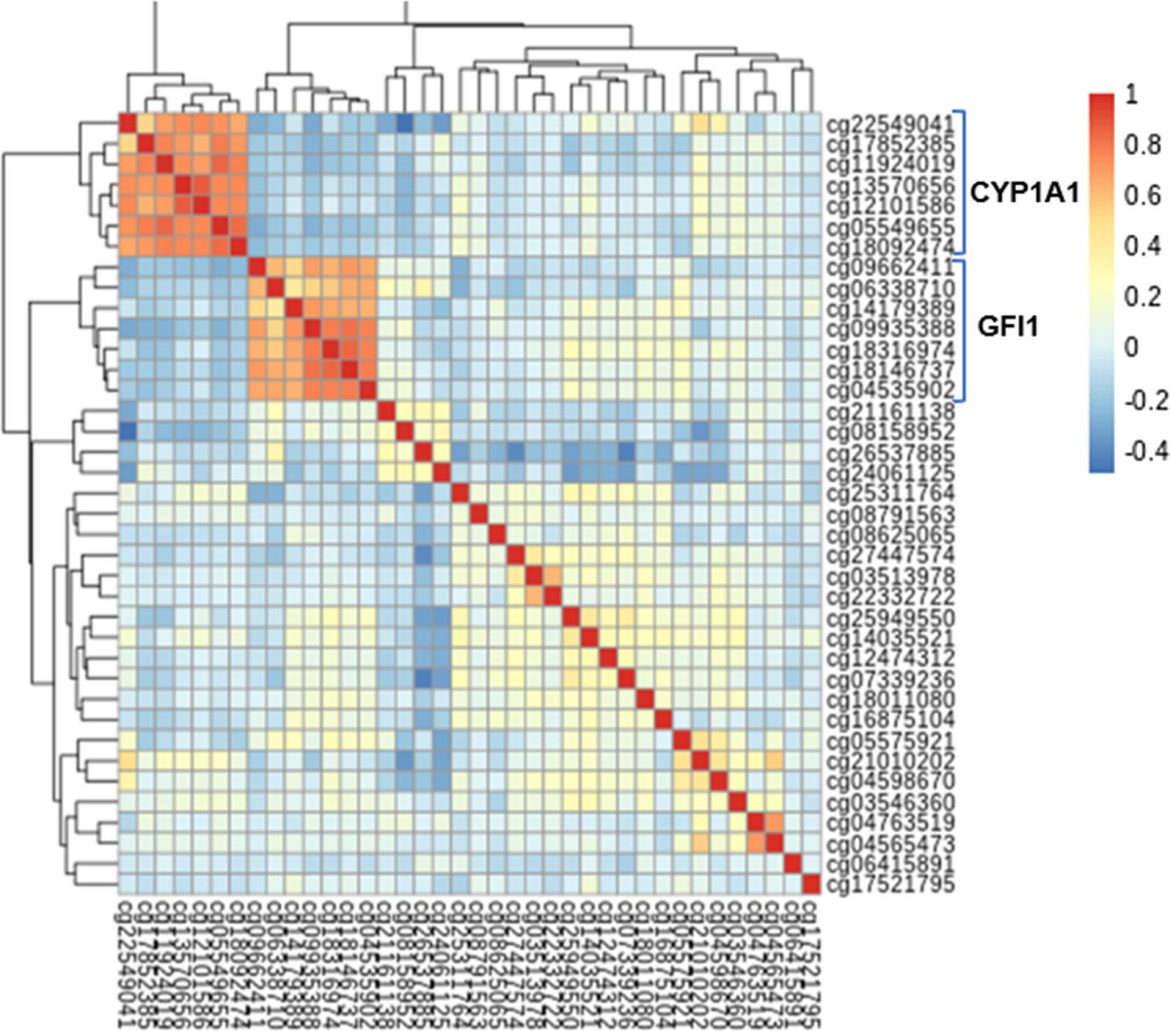
Heatmap of the correlation between the 38 significant cord blood DNA CpG sites associated with maternal smoking in 954 mother–newborn pairs in the Boston Birth Cohort

Figure 1 shows the Manhattan plot and Quantile–Quantile plot of the association between maternal smoking and cord blood DNAm. At a false discovery rate (FDR) < 0.05 as the genome-wide significance cutoff, we identified 38 CpG sites with altered DNAm in newborns exposed to maternal smoking compared to unexposed newborns. Of those, 17 CpG sites were mapped to four previously reported genes: *growth factor independent 1 transcriptional repressor (GFI1), Aryl-hydrocarbon receptors repressor (AHRR*), *cytochrome P450, family 1, member A1 (CYP1A1), and contactin associated protein 2 (CNTNAP2)*. Additional file 1: Table 1 presents details on the 38 CpG sites in terms of their chromosomal positions, corresponding gene names, their location within a gene, coefficients of DNAm association with smoking using inverse-normal transformed beta values, nominal *P* values, and FDR. The CpG sites in the *GFI1*, *AHRR and CNTNAP2* genes were significantly hypomethylated as indicated by the negative coefficients, and CpG sites in the *CYP1A1* gene were hypermethylated as indicated by the positive coefficients in relation to maternal smoking. We then explored the inter-correlations among the 38 CpG sites associated with maternal smoking via heatmap, as shown in Fig. 2. We found that CpG sites in the *GFI1* and *CYP1A1* genes were highly correlated. We used factor analysis to create a composite score to represent highly correlated CpG sites in the *GFI1* and *CYP1A1* genes (named as *GFI1* gene score and *CYP1A1* gene score, respectively), which were applied in the next step's gene-based mediation analysis.

As a sensitivity analysis, when maternal smoking was analyzed as an ordered three-categorical variable (0 = never, 1 = quitting during pregnancy; 2 = continuous smoking, Additional file 1: Fig. 1), we noticed that there was an inverse and dose–response relation between maternal smoking and DNAm as represented by the *GFI1* gene score (Panel A) and DNAm of cg05575921 in the *AHRR* gene (Panel B), with the lowest DNAm among newborns exposed to continuous smoking; and the same pattern was observed for newborn birthweight (Panel D). In contrast, there was a positive association between maternal smoking categories and DNAm as represented by the *CYP1A1* gene score (Panel C), with the highest DNAm among newborns exposed to continuous smoking. Given the limited sample size, our subsequent analyses were limited to the binary smoking categories: never vs. ever smoking. We have further performed EWAS with maternal smoking, stratified by maternal race (Blacks vs nonBlacks) (Additional file 1: Fig. 2; Additional file 1: Table 2). Of note, the main EWAS results for the overall sample appeared to be driven by Blacks. We found that a CgG site (cg05549655) in the *CYP1A1* gene was significantly associated with maternal smoking in both the Black and non-Black group. Similarly, CpGs sites in the other two known genes (*GFI1, AHRR*) also tended to have comparable associations with maternal smoking in both groups, although the associations in non-Blacks were no longer significant after FDR adjustment. There were also some CpGs sites which had associations with maternal smoking in Blacks only, as summarized in Additional file 1: Table 2. We have also tested smoking × race interactions on epigenome-wide DNAm, and we found only 1 significant interaction with CpG site cg14650464 (the *FANCC* gene), but this CpG site showed no association with birthweight (data not shown).

### Mediation effects of cord blood DNAm on the smoking—birthweight relationship

As illustrated in Additional file 1: Fig. 3, our data met the prerequisite for mediation analyses. Newborns with *in-utero* exposure to maternal smoking had 258 g lower birthweight than newborns without exposure (*P* < 0.001). Table 2 presents the associations between the identified 38 smoking-associated CpG sites and birthweight, with adjustment of gestational age and other covariates in the models. We found that a total of 8 CpG sites, in the *GFI1*, *AHRR*, and *CYP1A1* genes, had significant associations with birthweight at *P* < 0.0013 (Bonferroni correction for 38 tests). We also performed statistical tests for DNAm × sex and DNAm × race (Black vs non-Black) interactions on birthweight, respectively, but did not detect any significant interactions (results not shown).

**Table 2:** Association of newborn birthweight with 38 CpG sites and gene methylation scores that were significantly associated with maternal smoking during pregnancy in 954 mother–newborn pairs from the Boston Birth Cohort

Gene	CpG*	Associations with birthweight^a^		
		Beta	SE	*P*
*GFI1*	cg09662411	62.6	15.1	3.66E−05^#^
	cg06338710	36.5	16.1	0.020
	cg18146737	65.5	14.9	1.29E−05^#^
	cg18316974	60.4	15.0	6.37E−05^#^
	cg04535902	66	16.1	4.72E−05^#^
	cg09935388	68.6	15.5	1.09E−05^#^
	cg14179389	48.7	15.5	0.002
	GFI1 gene score	71.5	15.6	5.17E−06^#^
*AHRR*	cg05575921	92.0	15.4	3.00E−09^#^
	cg21161138	23.4	15.6	0.135
*CYP1A1*	cg05549655	− 59.1	17.6	7.97E−04^#^
	cg17852385	− 36.7	17.8	0.039
	cg13570656	− 50.4	16.7	0.003
	cg12101586	− 47	16.1	0.004
	cg22549041	− 32.9	16.1	0.041
	cg11924019	− 46.6	17.9	0.010
	cg18092474	− 56.5	17.2	0.001^#^
	CYP1A1 gene score	− 61.3	18.0	6.84E−04^#^
*DHRS3*	cg03546360	24.6	16.3	0.155
*PRPF38B*	cg18011080	21.5	15.6	0.169
*MEIS1*	cg08791563	23.1	15.6	0.140
*ST3GAL5*	cg26537885	2.54	17.8	0.886
*GARS*	cg16875104	17.8	16.3	0.274
*CNTNAP2*	cg25949550	6.2	15.7	0.694
*FBXO16*	cg12474312	− 3.8	15.6	0.808
*POLR1E*	cg14035521	47.4	15.4	0.002
*GCNT3*	cg25311764	− 41.2	15.5	0.008
*ZNF747*	cg17521795	16.3	15.4	0.291
*CDH2*	cg03513978	42.8	15.2	0.005
	cg22332722	22.4	15.4	0.146
*ZNF835*	cg08158952	20.5	14.9	0.169
*ATP9A*	cg07339236	31.3	17.0	0.065
*UFD1L*	cg08625065	10.4	16.2	0.518
Intergenic CpGs	cg06415891	− 29.4	16.0	0.067
	cg21010202	14.7	15.5	0.343
	cg04763519	− 4.3	15.8	0.786
	cg04565473	5.31	15.5	0.731
	cg04598670	21.5	15.3	0.160
	cg27447574	3.3	16.3	0.843
	cg24061125	− 19.7	14.9	0.187

^a^Adjusted for maternal age, education level, race, parity, pre-pregnancy BMI, alcohol consumption, gestational age at delivery, pre-gestational/gestational diabetes, child sex, cord blood cell compositions (CD8, CD4, NK, B cell, monocytes, granulocytes, nucleated red blood cells)

^*^For each CpG, inverse-normal transformed *ß*-value was analyzed

^#^Statistically significant based on Bonferroni correction (0.05/38 = 0.0013)

We then used the VanderWeele-Vansteelandt approach to estimate the mediation effect of each single CpG on the maternal smoking-newborn birthweight association. Additional file 1: Table 3 presents the mediation results for all 38 CpG sites associated with smoking. The mediation effect of individual CpG sites ranged from 0.5 to 44.6% of the total smoking-birthweight association. Of note, aside from the CpG sites in the *GFI1*, *AHRR* and *CYP1A1* genes, none of the rest CpG sites were both significantly associated with birthweight and had significant mediation effect. Therefore, the subsequent combined mediation analysis was limited to the *GFI1, AHRR,* and *CYP1A1* genes. When the *GFI1* gene score and *CYP1A1* gene score were analyzed instead of single CpG site, the mediation percentage was 44.6% for the *GFI1* gene and 29.8% for the *CYP1A1* gene, respectively (Table 3). The combined mediation effect of the three genes (*GFI1 gene score, AHRR cg05575921, CYP1A1 gene score*), estimated via the structural equation model, explained 67.8% of the total smoking effect on birthweight (Table 3).

**Table 3:** Mediation effect of single CpG, single gene score, and combined mediation effect of the *GFI1, AHRR*, and *CYP1A1* genes on the maternal smoking-newborn birthweight association in 954 mother–newborn pairs from the Boston Birth Cohort

Gene/CpG	Mediated effect (VanderWeele-Vansteelandt Approach)			
	Beta	SE	*P*	% Mediated
*GFI1* gene score	− 70.1	24.0	0.004	44.6
*AHRR* CpG cg05575921	− 61.7	28.3	0.029	38.5
*CYP1A1* gene score*	− 47.9	15.5	0.002	29.8

Model parameters	Mediation effect by structural equation model (simultaneous consideration of the 3 genes)		
	Beta	SE	*P*
Direct effect of maternal smoking	− 50.4	42.8	0.239
Indirect effect via *GFI1* gene score	− 31.3	11.6	0.007
Indirect effect via *AHRR* CpG cg05575921	− 55.2	13.3	< 0.001
Indirect effect via *CYP1A1* gene score	− 18.6	8.5	0.022
Sum of indirect effect (or mediation effect)	− 106.0	18.3	< 0.001
Total effect of maternal smoking	− 156.4	40.7	< 0.001
Percent mediated	67.8%		

All models adjusted for maternal age, education level, race, parity, pre-pregnancy BMI, alcohol consumption, pre-gestational/gestational diabetes, gestational age, child sex, cord blood cell compositions (CD8, CD4, NK, B cell, monocytes, granulocytes, nucleated red blood cells)

### Subgroup analyses

As shown in Additional file 1: Tables 4–7, we performed sex- and race-specific subgroup mediation analyses and found that the percentage mediation by the combination of three genes (*GFI1, AHRR, CYP1A1*) was 79.8% in males (*n* = 504); 52.3% in females (*n* = 450), 79.6% in Blacks (*n* = 679), and 46.1% in non-Blacks (*n* = 275). We also tested DNAm × sex and DNAm × race (black vs non-black) interactions on birthweight, respectively. However, we did not detect any significant DNAm × sex or DNAm × race interactions (results not shown).

## Discussion

Our study has made following contributions to the scientific literature with regards to *in-utero* exposure to maternal smoking, epigenetic modification, and offspring birthweight. This study extends previous work, all based on the Illumina Infinium Methylation27 or 450 BeadChip, [5, 6, 20] which were biased toward promoter regions and missed more dynamic methylation sites including the enhancers. To our knowledge, this is by far the largest EWAS using the latest Illumina Infinium MethylationEPIC BeadChip (with over 850 K CpG sites) among a population of predominantly urban, low-income, Black mother–newborn pairs in the US. We replicated CpG sites/genes (*GFI1, AHRR, CYP1A1,* and *CNTNAP2*) that were previously found to be associated with maternal cigarette smoking, mostly in European or non-Black populations [4, 5], suggesting shared associations by different ethnic population. As shown in Additional file 1: Table 1, we also identified additional significant genes, including *DHRS3* (cg03546360), *PRPF38B* (cg18011080), *MEIS1* (cg08791563), *ST3GAL5* (cg26537885), *GARS* (cg16875104), *FBXO16* (cg12474312), *POLR1E* (cg14035521), *GCNT3* (cg25311764), *ZNFZ47* (cg17521795), *CDH2* (cg03513978, cg22332722), *ZNF835* (cg08158952), *ATP9A* (cg07339236), and *UFD1L* (cg08625065)*.* However, except for *GFI1, AHRR*, and *CYP1A1*, none of the other genes stood out as having an FDR significant association with newborn birthweight. Although we cannot dismiss their importance in DoHad, they may act through different mechanisms (rather than altered fetal growth) on health and disease later in life.

For the first time, we quantitatively estimated the degree to which smoking-related alterations in cord blood DNAm CpG sites individually and collectively mediate the smoking-birthweight association. We achieved this by applying advanced mediation analyses, including the VanderWeele-Vansteelandt method for a single mediator and structural equation model for multiple mediators. Under the VanderWeele-Vansteelandt model, we found that 8 CpG sites in the three known genes (*GFI1, AHRR,* and *CYP1A1*) were significantly associated with newborn birthweight and substantially mediated the smoking-birthweight association. Under the structural equation model, the combined mediation effect of the *GFI1, AHRR,* and *CYP1A1* genes explained 67.8% of the smoking-birthweight association. There were considerable variations in the degree of mediation across the subgroups, with the mediation effect appears to be stronger in males (79.8%) than in females (52.3%), and stronger in Blacks (79.6%) than in non-Blacks (46.1%). If our mediation results can be further confirmed, it would imply that modification of DNAm is likely a very important biological pathway by which maternal smoking impairs fetal growth and, perhaps, long-term health.

Although we cannot interpret the significant mediation as a causal relationship, our findings are robust and biologically plausible. Consistent with previous literature, we have found strong and consistent evidence that maternal smoking during pregnancy can significantly alter fetal DNAm. As shown in Additional file 1: Fig. 1, there was a dose–response relationship between fetal CpG methylation levels and maternal smoking categories (never, quitter, and continuous smoking). We have replicated CpG sites in genes that have important biological functions [4–6, 19]. For example, *GFI1* is involved in diverse developmental processes, *AHRR* plays a key role in the aryl hydrocarbon receptor signaling pathway, and *CYP1A1* is involved in phase I metabolism, which regulates the detoxification of the components of tobacco smoke. In the future, by integrating genotyping data in mothers and infants, we will have the potential to test the causal relationship between maternal smoking and cord DNAm, and between cord DNAm and birthweight, via two-step mendelian randomization approaches [22].

Prior to this current study, there was only a limited number of studies on cord blood DNAm mediation of maternal smoking and newborn birthweight, and all of those were conducted in European populations. An early study [20] focused on 129 Dutch children exposed to maternal smoking versus 126 unexposed to maternal and paternal smoking, using the Illumina 450 K array and traditional regression models. It showed a significant mediation effect of DNAm in the *GFI1* and *NEUROG1* genes on the smoking—birthweight relationship. A more recent study [21] in 282 German children, using the Illumina 450K array, also showed evidence of cord blood DNAm in the *PIM1*, *CNTNAP2*, and *ITGB7* genes mediating the smoking-birthweight association, while no mediation effects were reported for DNAm in the *AHRR*, *GFI1* and *CYP1A1* genes. Using different sources of bio-samples, a recent study in a European sample (*n* = 441) [23] examined placental DNAm in the *MDS2*, *PBX1*, *CYP1A2*, *VPRBP*, *WBP1L*, *CD28*, and *CDK6* genes as mediators of maternal smoking on birthweight and demonstrated significant mediation effects. A more recent study in a Danish case–control study of autism examined neonatal DNAm (blood sample obtained about 1 week after birth) and again found that maternal smoking was significantly associated with both birthweight and neonatal DNAm, including CpG sites in *GFI1 and AHRR genes *[24] 

In comparison, our study, in a larger birth cohort composed of predominantly Black mother–infant pairs, confirmed DNAm mediation effects of CpG sites in the *AHRR, GFI1* and *CYP1A1* genes, and indicated that such effects tended to be stronger in Blacks. Of note, the main EWAS findings with maternal smoking in the overall sample appear to be driven by the black sample. Our tests for DNAm × race interactions on birthweight were not significant. It is unclear whether there are true differences between the Black and non-Black due to sample size constraint, especially in the non-Black subset. Our stratified analyses may be underpowered, and the findings remain to be confirmed in future studies and their implications remain to be understood. It is known that DNAm profiles are sex-specific as early as at birth. However, few studies have performed sex-stratified mediation analyses, which may be partly due to a limited sample size in previous studies. The study by Witt et al. indicated that the CpG site in *CNTNAP2* had a more pronounced effect in male newborns. Consistently, our results showed that the DNAm mediation effects on smoking—birthweight relationships were stronger in males, suggesting that such mediation effect may be sex-dependent. However, our interaction tests for DNAm × sex were not significant. Future studies should further examine potential sex and racial differences in the DNAm and birthweight associations.

Our findings, especially the significant mediation by cord blood DNAm, if further confirmed, have important implications for research and clinical and public health practice. Beyond this study, the interplay of maternal smoking, fetal DNAm and birthweight could serve as a prototype to understand the impact of other early life environmental exposures on the developing fetus and their underlying biological mechanisms. Prospective birth cohort studies with long-term postnatal follow-up will offer important opportunities to further investigate the possibility that altered fetal DNAm may have a long-lasting impact on the exposed child’s health over the life course.

Our study has following limitations. First, it lacks a replication sample, and DNAm profiles were not validated using other independent technologies. However, we replicated CpG sites in the *GFI1*, *AHRR, CYP1A1,* and *CNTNAP2* genes which are well-known to be associated with cigarette smoking [4–6, 19]. We further demonstrated that the altered CpG sites in the first three genes were associated with newborn birthweight and significantly mediated the maternal smoking-newborn birthweight association, supporting the biological plausibility of our findings. Second, maternal smoking in this study is self-reported and may be subject to reporting bias. We validated this variable by measuring known metabolites of nicotine in a subset (*n* = 630), including cotinine and hydroxy-cotinine, in both maternal plasma and cord blood plasma (direct evidence of fetal exposure). As shown in Additional file 1: Fig. 4, there are distinct differences in the distributions of these metabolites by maternal self-reported smoking during pregnancy, and their levels were the highest in mothers and newborns with self-reported continuous smoking during pregnancy. However, there are observed overlaps in the distribution between self-reported never smoking vs. ever smoking which could be due to several reasons: (1) maternal smoking is under reported; (2) Plasma cotinine and hydroxy-cotinine can only reflect recent maternal smoking (half-life 1–3 days), which may explain partly that some smoking mothers had extremely low cotinine levels, which is possible if they did not smoke during or after delivery; and (3) exposure to passive smoking, which may partly explain why some never-smoking mothers have relatively high levels of cotinine. Although both self-reported smoking and biomarkers have limitations, to a large degree, our data lent support that self-reported smoking in our study is overall a reliable indicator of maternal smoking status. Third, despite being relatively large, our study may still be underpowered to identify CpG sites with smaller effect sizes. We also were unable to further explore observed differences in the percentage of mediation by DNAm between males and females, between Black and non-Black groups, and between quitters and continuous smokers due to limited sample sizes. Fourth, this study was conducted in a US predominantly urban, low-income, multiethnic population, which is both a strength (understudied population) and a weakness (less generalizable). The current study sample is a subset of the Boston Birth Cohort. As shown in Additional file 1: Table 8, overall, the included and excluded samples were comparable for the baseline demographic and clinical variables, except that the included sample of the current study had a higher percentage of Black participants. We are uncertain whether the higher percentage of mediation effect in Blacks was due to a larger sample size of Black participants compared to non-Black participants or if there was a real racial difference. Nevertheless, our overall findings on the effect of maternal smoking on birthweight and cord blood DNAm are remarkably consistent with previous studies across diverse populations, [4–6, 19] lending support that the smoking effect on fetal growth and DNAm is likely universal. Fifth, with increasing availability of high-dimensional epigenomic data, there is growing interest to quantify the extent to which the effect of environmental factors on health is mediated by multiple epigenetic marks or on a genome-wide scale. As underscored by a recent commentary [25], “given the lack of an overarching validated framework and the generally complex causal structure of high-dimensional data, the analysis of high-dimension mediation currently requires great caution and effort to incorporate a priori biological knowledge”, more studies are needed to confirm our findings related to the degree of mediation and whether it is causal although our mediation analysis followed a priori biology and hypotheses. Finally, given the epigenome is at the interface of gene and environment, future studies are needed to add the genome to the puzzle and to further delineate the complex interplay of genome, epigenome, and environmental effects on health outcomes [26].

## Conclusion

In this predominantly US urban, low-income multiethnic sample, using the latest Illumina Infinium MethylationEPIC BeadChip, we identified a total of 38 differentially methylated CpG sites with genome-wide significance (FDR < 0.05) associated with maternal smoking. Of those, 8 CpG sites in the *GFI1, AHRR,* and *CYP1A1* genes were Bonferroni significantly associated with newborn birthweight and their combined mediation effect explained 67.8% of the smoking-birthweight association. Our findings raise the possibility that altered fetal DNAm may not only serve as a biomarker of *in-utero* smoking exposure (quitter or continuous), but also may represent an important biological pathway by which maternal smoking impairs fetal growth, and, perhaps, even long-term health outcomes. Beyond this current study, the methodologies we used may serve as a prototype for future investigations of other *in-utero* adverse environmental exposures on the developing fetus and health across the life course.

## Methods

### Study population

Additional file 1: Fig. 5 presents the flow chart of the study participants. This study included 954 mother–newborn pairs from the Boston Birth Cohort (BBC), a US predominantly urban low-income, Black population. The BBC was initiated in 1998 with rolling enrollment at the Boston Medical Center, in Boston, MA, as detailed elsewhere [3]. In brief, mothers who delivered singleton live births at the Boston Medical Center were invited to participate shortly after giving birth. The BBC is enriched by preterm (< 37 weeks of gestation) and low birthweight (< 2500 g) births due to by design over-sampling at enrollment. Pregnancies that were a result of in vitro fertilization, multiple gestations (e.g., twins, triplets), fetal chromosomal abnormalities or major birth defects or preterm birth due to trauma were excluded. After mothers gave written informed consent, they were enrolled and asked to complete a standard questionnaire interview on maternal socio-demographic characteristics, lifestyle including smoking and alcohol consumption, diet, and reproductive and medical history. Maternal and newborn clinical information including birth outcomes were obtained from their medical records. The study protocol has received initial and annual continuation approval by the Institutional Review Boards of Boston Medical Center and the Johns Hopkins Bloomberg School of Public Health. Additional file 1: Table 8 presents characteristics of the 954 pairs enrolled in this study compared to the rest mother–newborn pairs (*n* = 7555) in the BBC.

### Definition of maternal smoking, the primary exposure

Maternal smoking during pregnancy was defined based on maternal questionnaire interview at enrollment: ‘(1) in the six months before you found out you were pregnant, did you smoke/use tobacco?’; (2) ‘Did you smoke/use tobacco in the first three months of pregnancy?’; (3) ‘Did you smoke/use tobacco in the middle three months of pregnancy?’; and (4) ‘Did you smoke/use tobacco in the last three months of pregnancy?’. We defined ‘maternal smoking during pregnancy’ as those mothers who answered ‘yes’ to any of the above questions and defined ‘non-smoking’ mothers as those mothers who answered ‘no’ to all the above questions. Additional sensitivity analyses were performed to further divide maternal smoking into “Quitters—who stopped smoking since the 1st or 2nd or 3rd trimester” versus “Continuous smokers—who continued to smoke during the entire pregnancy”. In a subset (*n* = 630), we validated self-reported maternal smoking by measuring known metabolites of nicotine, including cotinine and hydroxy cotinine, in both maternal plasma and cord blood plasma (direct evidence of fetal exposure), as shown in the Additional file 1: Fig. 4.

### Definition of important covariates

Other important covariates that were adjusted in the analysis included: maternal age at delivery, parity (not including the index pregnancy, 0 versus 1 or more), maternal education (high school or less versus some college or more), maternal self-reported race (Black versus non-Black): Black included self-reported Black (African American and Haitian) and non-Black included white and Hispanic, maternal alcohol consumption during pregnancy (never versus ever), maternal pre-pregnancy body mass index (BMI), maternal pregestational/gestational diabetes (yes versus no), child’s sex (female versus male), and gestational age (GEAA) at birth. As detailed in our earlier publication[3] in the BBC, GEAA was estimated using an established algorithm based on both last menstrual period (LMP) and the result of early ultrasound (U/S, < 20 weeks of gestation) to maximize accuracy and minimize missing data. In this cohort, about 70% of the mothers had early U/S and corresponding percentage of early U/S among never vs. ever smoking mothers were 72.1% vs. 68.5% (*P* = 0.398). Most of the covariates have been previously defined and published [27].

#### DNAm profiling in cord blood and quality control steps 

Cord blood was obtained by the trained nursing staff of the labor and delivery service; the quality of the DNA samples has been demonstrated in our previous studies using the Illumina BeadChip [27]. A total of 963 cord DNA samples (plus 21 replicates) were sent to the University of Minnesota Genomics Center for genome-wide DNAm profiling, using the Illumina Infinium MethylationEPIC BeadChip (850K). With this platform, DNAm profiles for a total of 865,859 CpG sites (at locations of cytosines followed by guanine) were successfully generated and a β-value for each CpG site examined was reported, ranging from 0–1.0, to signify the percentage of DNAm at each CpG site. Systematic quality control steps were performed using existing analytic pipelines with R/Bioconductor package ‘minfi’ [28]. Briefly, with a raw intensity file (.idat) for each sample, first, the 850K control probes were examined to assess bisulfite conversion, extension, hybridization, staining, specificity and other factors. Second, the median for both Meth and Unmeth signals for each array were computed and displayed in a scatter plot to identify outlier samples with low intensity. Using a median log_2_ intensity value < 9 as the cutoff, 1 sample that appeared to be an outlier was removed from subsequent analyses. A sensitivity analysis, performed after removing an additional 12 samples with median log2 intensity < 10, yielded very similar results. Third, correlations for the duplicates were computed among the 21 pairs of duplicates. Fourth, although this report did not include maternal DNAm, for DNA methylation profiling, both maternal samples (*n* ~ 420 samples) and cord DNA samples were randomly placed in each 96-well DNA plate. Lab personnel were blinded to the sample placement. Data QC steps were performed in cord samples, along with mother–child dyad and newborn sex information. Furthermore, multi-dimensional scaling (MDS) plots were used to evaluate outlier samples and confirm male cord blood samples, female cord blood samples and maternal blood samples, which clustered separately, as expected. Seven samples whose DNAm data-derived sex values were inconsistent with sex as documented in medical records were identified and removed from the subsequent analyses. Fifth, sample-wise missing rates were calculated and one sample with > 2% missing rate was removed. At the locus level, 4193 CpG sites that had a detection *P* value > 0.01 in more than 5% of the samples were removed. An additional 140,271 CpG sites were removed due to the following reasons: having an annotated SNP at the measured or extension site or that overlapped with the probe, and/or that potentially cross-hybridized to other genomic locations [29]. Then, we applied the single-sample Noob (ssNoob) methods via “preprocessnoob” function for background and dye bias correction [30], and then performed quantile normalization in “preprocessQuantile” to normalize type 1 and type II probes. We additionally removed 16,843 CpG sites in the sex chromosomes. These filters resulted in high quality DNAm data for 704,552 CpG sites in 954 samples for subsequent analyses.

### Statistical methods

Summary statistics were performed to compare the demographic and clinical characteristics of newborns who were exposed to maternal smoking during pregnancy versus those who were not exposed, using the chi-square test for categorical variables and ANOVA for continuous variables. We applied the following analytical methods to address the study objectives. All statistical analyses were performed using R (version 4.0.2; R Foundation for Statistical Computing).

### Identification of differentially methylated sites

We investigated differentially methylated sites in cord blood that were associated with maternal smoking, using the ‘limma’ package [31]. To address the skewed distribution and potential outliers or bimodal distribution of the original β-values, we applied an inverse normal transformation and obtained inversely normalized β-values for each CpG. To account for potential batch effects, models were additionally adjusted with calculated surrogate variables (SV) using the ‘SmartSVA’ package [32]. A total of 62 SV variables were generated that were then adjusted in the subsequent analyses. We fitted a linear regression model with the inversely-normalized β-value at each CpG site as the outcome and maternal smoking (1 = ever, 0 = never) as the independent variable, adjusting for potential confounders including maternal age, education level, race, parity, pre-pregnancy BMI, alcohol consumption, gestational age, pregestational/gestational diabetes, child sex, and SV variables. We also adjusted for estimated cord blood cell composition, which was calculated using the estimateCellCounts() function in the ‘minfi’ package, where the distribution of each cell type, including CD4 + , CD8 + T cells, B cells, monocytes, granulocytes, natural killer cells, and nucleated red blood cells (specific to cord blood), was inferred for each cord blood sample, based on external cord blood reference DNA methylation signatures of the constituent cell type from the Illumina Infinium Methylation450 BeadChip [33]. The false discovery rate (FDR)[34] was applied to correct for multiple testing, with FDR < 0.05 as the genome-wide significance cutoff. Stratified analyses by maternal race were also performed.

### Mediation analyses

Additional file 1: Fig. 3 illustrates our sequential analytical methods to dissect the interplay of maternal smoking, cord blood DNAm, and newborn birthweight. As a first step, we examined whether the significant smoking—associated CpG sites, as identified above, are associated with newborn birthweight adjusting for GEAA, maternal age, education level, race, parity, pre-pregnancy BMI, alcohol consumption, pre-gestational/gestational diabetes, child sex, cord blood cell compositions; thus, we focused on their effect on fetal growth. Scatter plots of birthweight versus each CpG methylation site were first inspected, which supports the use of linear regression models to assess the adjusted associations between each CpG (as the exposure) and newborn birthweight (as the outcome). Then, for those CpG sites that were associated with maternal smoking, we estimated the degree to which maternal smoking-associated CpG alterations, individually and collectively, mediate the maternal smoking-birthweight association. First, we applied the VanderWeele-Vansteelandt method[35] for assessing mediation and interaction effect by each CpG on the smoking-birthweight association, implemented by the R ‘medflex’ package. Second, for genes with multiple significant CpG sites, we examined their correlations using a heatmap and then, used factor analyses [36] to create a composite score to represent highly correlated CpG sites in a gene, implemented by R ‘psych’ package. We then applied the composite score to perform gene-based mediation analysis using the R ‘medflex’ package. Finally, we estimated a combined mediation effect of the major significant genes, using the structural equation modeling approach [37], implemented by the R ‘lavaan’ package. The significance was based on *P* values provided by the VanderWeele-Vansteelandt and the structural equation models, respectively.

### Sensitivity analyses

To further explore if the above mediation estimation differed by fetal sex and maternal race, we performed sex-specific and race-specific mediation analyses to estimate percent mediation by cord DNAm on the smoking-birthweight association, using similar methods as described above. We also tested DNAm × sex and DNAm × race (Black vs. non-Black) interactions on birthweight.

## Supplementary Information

**Additional file 1: Table 1**. Genome-wide DNA methylation association study identified 38 CpG sites significantly associated with maternal smoking during pregnancy in 954 mother–newborn pairs from the Boston Birth Cohort. **Additional file 1: Table 2**. Genome-wide DNA methylation association study with maternal smoking during pregnancy: Comparison of significant CpGs among total sample, Blacks only, and non-Blacks only. **Additional file 1: Table 3**. Methylation mediation effect of each of the 38 single CpG sites on the maternal smoking-newborn birthweight association in 954 mother–newborn pairs from the Boston Birth Cohort. **Additional file 1: Table 4**. Single CpG, single gene score, and combined multiple gene mediation analysis on the maternal smoking—newborn birthweight association, among 504 male newborns in the Boston Birth Cohort. **Additional file 1: Table 5**. Single CpG, single gene score, and combined multiple gene mediation analysis on the maternal smoking—newborn birthweight association, among 450 female newborns in the Boston Birth Cohort. **Additional file 1: Table 6**. Single CpG, single gene score, and combined multiple gene mediation analysis on the maternal smoking—newborn birthweight association, among 679 Black newborns in the Boston Birth Cohort. **Additional file 1: Table 7**. Single CpG, single gene score, and combined multiple gene mediation analysis on the maternal smoking—newborn birthweight association, among 275 non-Black newborns in the Boston Birth Cohort. **Additional file 1: Table 8**. Characteristics of mother–newborn pairs included (N = 954) versus excluded (N = 7555) from this current study. **Additional file 1: Figure 1**. Illustration of the relationship between maternal smoking categories (0 = never, 1 = quitter, 2 = current) and GFI1 gene score (Panel A), AHRR cg05575921 (Panel B), and CYP1A1 gene score (Panel C); and between smoking categories (0 = never, 1 = quitter, 2 = current) and birthweight (Panel D), in 954 mother–newborn pairs from the Boston Birth Cohort. **Additional file 1: Figure 2**. Manhattan and Q-Q plots for the EWAS analyses in cord blood in associations with maternal smoking, in Black (Fig 2A) and non-Black subset (Figure 2B). Grey dotted line represents the epigenome-wide significance cut-off after Bonferroni correction, and the red dotted line represents the epigenome-wide significance cut-off with FDR< 0.05; Genes in red represent known genes associated with smoking as identified in previous studies. **Additional file 1: Figure 3**. An illustration of our sequential analytical methods to dissect the interplay of maternal smoking (X), cord blood DNA methylation (M: mediator), and newborn birthweight (Y), where C’ represents the direct effect of X on Y. **Additional file 1: Figure 4**. Distribution of cotinine/hydroxy-cotinine by smoking status: Panel A: cord plasma cotinine/hydroxy-cotinine by maternal smoking status; Panel B: maternal plasma cotinine/hydroxy-cotinine by maternal smoking status. **Additional file 1: Figure 5**. Flowchart of Study Participants.

## Data Availability

The datasets supporting these findings are not publicly available. Instead, the datasets used and/or analyzed for the current study are available from the corresponding author on reasonable request and after Institutional Review Board review and approval.

## Abbreviations

*AHRR*: Aryl-hydrocarbon receptors repressor
BBC: Boston Birth Cohort
BMI: Body mass index
*CNTNAP2*: Contactin associated protein 2
CpG sites: DNA methylation occurs predominantly at locations of cytosines followed by guanine residues (CpG).
*CYP1A1*: Cytochrome P450, family 1, member A1
DoHaD: Developmental origins of health and disease
DNA: Deoxyribonucleic acid
DNAm: DNA methylation
EWAS: Epigenome-wide association study
FDR: False discovery rate
GEAA: Gestational age at delivery
*GFI1*: Growth factor independent 1 transcriptional repressor
SNP: Single nucleotide polymorphism
