# Supplementary material for: DNA methylation mediates the effect of maternal smoking on offspring birthweight: a birth cohort study of multi-ethnic US mother–newborn pairs
Source: Clin Epigenetics. 2021 Mar 4;13:47. doi: 10.1186/s13148-021-01032-6 (PMC7931602; doi:10.1186/s13148-021-01032-6)
Supplement: Supplementary file 1 — Additional file 1: Table 1. Genome-wide DNA methylation association study identified 38 CpG sites significantly associated with maternal smoking during pregnancy in 954 mother–newborn pairs from the Boston Birth Cohort. Additional file 1: Table 2. Genome-wide DNA methylation association study with maternal smoking during pregnancy: Comparison of significant CpGs among total sample, Blacks only, and non-Blacks only. Additional file 1: Table 3. Methylation mediation effect of each of the 38 single CpG sites on the maternal smoking-newborn birthweight association in 954 mother–newborn pairs from the Boston Birth Cohort. Additional file 1: Table 4. Single CpG, single gene score, and combined multiple gene mediation analysis on the maternal smoking—newborn birthweight association, among 504 male newborns in the Boston Birth Cohort. Additional file 1: Table 5. Single CpG, single gene score, and combined multiple gene mediation analysis on the maternal smoking—newborn birthweight association, among 450 female newborns in the Boston Birth Cohort. Additional file 1: Table 6. Single CpG, single gene score, and combined multiple gene mediation analysis on the maternal smoking—newborn birthweight association, among 679 Black newborns in the Boston Birth Cohort. Additional file 1: Table 7. Single CpG, single gene score, and combined multiple gene mediation analysis on the maternal smoking—newborn birthweight association, among 275 non-Black newborns in the Boston Birth Cohort. Additional file 1: Table 8. Characteristics of mother–newborn pairs included (N = 954) versus excluded (N = 7555) from this current study. Additional file 1: Figure 1. Illustration of the relationship between maternal smoking categories (0 = never, 1 = quitter, 2 = current) and GFI1 gene score (Panel A), AHRR cg05575921 (Panel B), and CYP1A1 gene score (Panel C); and between smoking categories (0 = never, 1 = quitter, 2 = current) and birthweight (Panel D), in 954 mother–newborn pairs from the Boston Birth [file 13148_2021_1032_MOESM1_ESM.docx]

**Supplemental files**

**Supplemental Table 1.** Genome-wide DNA methylation association study identified 38 CpG sites significantly associated with maternal smoking during pregnancy in 954 mother-newborn pairs from the Boston Birth Cohort.

| **CpG** | **CHR** | **Position** | **Gene** | **Location** | **Beta*** | **P** | **FDR** |
| --- | --- | --- | --- | --- | --- | --- | --- |
| cg06415891 | 1 | 2467096 | N/A | N/A | 0.344 | 1.23E-06 | 0.026 |
| cg03546360 | 1 | 12675968 | *DHRS3* | Body | -0.33 | 8.55E-07 | 0.020 |
| cg09662411 | 1 | 92946132 | *GFI1* | Body | -0.532 | 8.82E-11 | 7.64E-06 |
| cg06338710 | 1 | 92946187 | *GFI1* | Body | -0.448 | 8.04E-09 | 3.77E-04 |
| cg18146737 | 1 | 92946700 | *GFI1* | Body | -0.555 | 9.76E-11 | 7.64E-06 |
| cg18316974 | 1 | 92947035 | *GFI1* | Body | -0.603 | 7.38E-13 | 1.30E-07 |
| cg04535902 | 1 | 92947332 | *GFI1* | Body | -0.525 | 4.35E-11 | 5.10E-06 |
| cg09935388 | 1 | 92947588 | *GFI1* | Body | -0.593 | 4.62E-13 | 1.08E-07 |
| cg14179389 | 1 | 92947961 | *GFI1* | Body | -0.463 | 5.96E-09 | 3.00E-04 |
| cg18011080 | 1 | 109234128 | *PRPF38B* | TSS1500 | -0.463 | 3.98E-09 | 2.15E-04 |
| cg08791563 | 2 | 66736054 | *MEIS1* | Body | -0.373 | 1.77E-06 | 0.035 |
| cg26537885 | 2 | 86100811 | *ST3GAL5* | Body | -0.252 | 2.14E-06 | 0.040 |
| cg05575921 | 5 | 373378 | *AHRR* | Body | -0.714 | 1.53E-26 | 1.08E-20 |
| cg21161138 | 5 | 399360 | *AHRR* | Body | -0.411 | 1.59E-08 | 7.02E-04 |
| cg21010202 | 6 | 1615843 | *N/A* | N/A | -0.328 | 5.08E-08 | 0.002 |
| cg04763519 | 6 | 1619094 | *N/A* | N/A | -0.41 | 1.59E-07 | 0.006 |
| cg04565473 | 6 | 1619162 | *N/A* | N/A | -0.423 | 8.94E-07 | 0.020 |
| cg16875104 | 7 | 30635889 | *GARS* | Body | -0.373 | 8.71E-07 | 0.020 |
| cg04598670 | 7 | 68697651 | *N/A* | N/A | -0.359 | 2.07E-09 | 1.21E-04 |
| cg25949550 | 7 | 145814306 | *CNTNAP2* | Body | -0.336 | 2.57E-07 | 0.008 |
| cg12474312 | 8 | 28348088 | *FBXO16* | TSS1500 | -0.369 | 2.16E-06 | 0.040 |
| cg14035521 | 9 | 37486469 | *POLR1E* | Body | -0.338 | 1.11E-06 | 0.0245 |
| cg27447574 | 12 | 75374516 | *N/A* | N/A | -0.322 | 1.40E-06 | 0.028 |
| cg24061125 | 14 | 97165167 | *N/A* | N/A | 0.294 | 8.95E-07 | 0.020 |
| cg25311764 | 15 | 59907427 | *GCNT3* | 5'UTR | 0.325 | 3.34E-07 | 0.009 |
| cg05549655 | 15 | 75019143 | *CYP1A1* | TSS1500 | 0.503 | 2.16E-13 | 7.60E-08 |
| cg17852385 | 15 | 75019188 | *CYP1A1* | TSS1500 | 0.403 | 3.49E-08 | 0.001 |
| cg13570656 | 15 | 75019196 | *CYP1A1* | TSS1500 | 0.33 | 2.07E-07 | 0.007 |
| cg12101586 | 15 | 75019203 | *CYP1A1* | TSS1500 | 0.351 | 2.74E-08 | 0.001 |
| cg22549041 | 15 | 75019251 | *CYP1A1* | TSS1500 | 0.314 | 7.67E-10 | 5.40E-05 |
| cg11924019 | 15 | 75019283 | *CYP1A1* | TSS1500 | 0.44 | 7.49E-11 | 7.54E-06 |
| cg18092474 | 15 | 75019302 | *CYP1A1* | TSS1500 | 0.474 | 6.28E-12 | 8.85E-07 |
| cg17521795 | 16 | 30545145 | *ZNF747* | Body | -0.384 | 2.20E-07 | 0.007 |
| cg03513978 | 18 | 25616805 | *CDH2* | TSS1500;Body | -0.391 | 4.06E-07 | 0.011 |
| cg22332722 | 18 | 25754857 | *CDH2* | Body | -0.394 | 1.22E-06 | 0.026 |
| cg08158952 | 19 | 57183016 | *ZNF835* | Body | -0.365 | 1.66E-07 | 0.006 |
| cg07339236 | 20 | 50312490 | *ATP9A* | Body | -0.425 | 9.16E-10 | 5.87E-05 |
| cg08625065 | 22 | 19465372 | *UFD1L* | Body | -0.368 | 2.70E-07 | 0.008 |

*For estimation of beta coefficient, DNA methylation (as the outcomes) was analyzed based on inverse-normal transformation of β values.

TSS: transcription starting site; 5’UTR: 5’ untranslated region.

**Adjusted for maternal age, education level, race/ethnicity, parity, pre-pregnancy BMI, alcohol consumption, gestational age, pregestational/gestational diabetes, child sex, cord blood cell compositions (CD8, CD4, NK, B cell, monocytes, granulocytes, nucleated red blood cells) and batch effect.

**Supplemental Table 2.** Genome-wide DNA methylation association study with maternal smoking during pregnancy: Comparison of significant CpGs among total sample, Blacks only, and non-Blacks only.

|  |  |  | Total^$^  (165 smokine vs 789 non-smoking) | | Blacks^+^  (113 smoking vs 566 non-smoking | | Non-Blacks^+^  (52 smoking vs 223 non-smoking | |
| --- | --- | --- | --- | --- | --- | --- | --- | --- |
| CpG | CHR | Gene | Beta | P^a^ | Beta | P^a^ | Beta | P^a^ |
| cg06415891 | 1 | N/A | 0.344 | 1.23E-06^a^ | 0.273 | 0.001 | 0.444 | 0.002 |
| cg00850612 | 1 | KCNAB2 | 0.156 | 0.011 | 0.378 | 4.05E-07^a^ | -0.207 | 0.073 |
| cg21681377 | 1 | DNAJC11 | -0.263 | 4.58E-05 | -0.374 | 2.05E-06^a^ | -0.074 | 0.545 |
| cg03546360 | 1 | DHRS3 | -0.330 | 8.55E-07^a^ | -0.366 | 9.16E-06 | -0.277 | 0.028 |
| cg09662411 | 1 | GFI1 | -0.532 | 8.82E-11^a^ | -0.597 | 8.48E-10^a^ | -0.611 | 2.46E-04 |
| cg06338710 | 1 | GFI1 | -0.448 | 8.04E-09^a^ | -0.488 | 1.09E-07^a^ | -0.591 | 2.81E-04 |
| cg18146737 | 1 | GFI1 | -0.555 | 9.76E-11^a^ | -0.642 | 3.57E-10^a^ | -0.577 | 0.001 |
| cg18316974 | 1 | GFI1 | -0.603 | 7.38E-13^a^ | -0.678 | 2.31E-11^a^ | -0.654 | 1.05E-04 |
| cg04535902 | 1 | GFI1 | -0.525 | 4.35E-11^a^ | -0.648 | 1.02E-11^a^ | -0.548 | 8.45E-04 |
| cg09935388 | 1 | GFI1 | -0.593 | 4.62E-13^a^ | -0.635 | 1.64E-10^a^ | -0.672 | 4.83E-05 |
| cg14179389 | 1 | GFI1 | -0.463 | 5.96E-09^a^ | -0.526 | 8.14E-08^a^ | -0.463 | 0.002 |
| cg18011080 | 1 | PRPF38B | -0.463 | 3.98E-09^a^ | -0.567 | 5.43E-09^a^ | -0.157 | 0.265 |
| cg08791563 | 2 | MEIS1 | -0.373 | 1.77E-06^a^ | -0.451 | 3.04E-06 | -0.290 | 0.049 |
| cg26537885 | 2 | ST3GAL5 | -0.252 | 2.14E-06^a^ | -0.163 | 0.010 | -0.275 | 0.010 |
| cg07638500 | 3 | MYLK | -0.205 | 7.13E-05 | -0.290 | 1.64E-06^a^ | -0.104 | 0.328 |
| **cg05575921** | **5** | **AHRR** | **-0.714** | **1.53E-26^a^** | **-0.753** | **2.99E-22^a^** | **-0.698** | **8.69E-07** |
| cg21161138 | 5 | AHRR | -0.411 | 1.59E-08^a^ | -0.545 | 5.48E-10^a^ | -0.132 | 0.336 |
| cg07136635 | 5 | ITGA1;PELO | -0.285 | 1.79E-04 | -0.494 | 4.58E-08^a^ | 0.076 | 0.606 |
| cg16619991 | 5 | ITGA1;PELO | -0.317 | 3.06E-05 | -0.481 | 1.83E-07^a^ | -0.031 | 0.824 |
| cg21010202 | 6 | N/A | -0.328 | 5.08E-08^a^ | -0.338 | 3.01E-06 | -0.439 | 3.61E-04 |
| cg04763519 | 6 | N/A | -0.410 | 1.59E-07^a^ | -0.419 | 1.09E-05 | -0.453 | 0.002 |
| cg04565473 | 6 | N/A | -0.423 | 8.94E-07^a^ | -0.461 | 1.16E-05 | -0.398 | 0.012 |
| cg20742318 | 6 | GCLC | 0.256 | 3.03E-04 | 0.443 | 1.20E-07^a^ | -0.098 | 0.469 |
| cg01976472 | 7 | N/A | -0.148 | 3.00E-06 | -0.189 | 7.34E-07^a^ | -0.121 | 0.047 |
| cg16875104 | 7 | GARS | -0.373 | 8.71E-07^a^ | -0.464 | 3.48E-07^a^ | -0.371 | 0.009 |
| cg04598670 | 7 | N/A | -0.359 | 2.07E-09^a^ | -0.330 | 3.51E-06 | -0.222 | 0.092 |
| cg25949550 | 7 | CNTNAP2 | -0.336 | 2.57E-07^a^ | -0.319 | 8.13E-05 | -0.456 | 4.18E-05 |
| cg12474312 | 8 | FBXO16 | -0.369 | 2.16E-06^a^ | -0.311 | 0.001 | -0.387 | 0.006 |
| cg21789008 | 8 | N/A | -0.252 | 6.76E-05 | -0.361 | 2.04E-06^a^ | -0.027 | 0.829 |
| cg14035521 | 9 | POLR1E | -0.338 | 1.11E-06^a^ | -0.358 | 3.39E-05 | -0.423 | 8.57E-04 |
| cg14650464 | 9 | FANCC* | -0.056 | 0.484 | 0.160 | 0.096 | -0.647 | 1.61E-05 |
| cg07496388 | 10 | ANK3 | 0.369 | 1.51E-05 | 0.524 | 5.45E-07^a^ | 0.238 | 0.111 |
| cg07246206 | 11 | SUV420H1 | -0.304 | 1.24E-05 | -0.398 | 1.01E-06^a^ | -0.028 | 0.841 |
| cg27447574 | 12 | N/A | -0.322 | 1.40E-06^a^ | -0.265 | 0.002 | -0.304 | 0.021 |
| cg24061125 | 14 | N/A | 0.294 | 8.95E-07^a^ | 0.210 | 0.004 | 0.374 | 5.86E-04 |
| cg25311764 | 15 | GCNT3 | 0.325 | 3.34E-07^a^ | 0.276 | 2.95E-04 | 0.379 | 0.003 |
| **cg05549655** | **15** | **CYP1A1** | **0.503** | **2.16E-13^a^** | **0.394** | **1.33E-06^a^** | **0.757** | **3.44E-09 ^a^** |
| cg17852385 | 15 | CYP1A1 | 0.403 | 3.49E-08^a^ | 0.407 | 3.74E-06 | 0.396 | 0.004 |
| cg13570656 | 15 | CYP1A1 | 0.330 | 2.07E-07^a^ | 0.324 | 2.52E-05 | 0.299 | 0.014 |
| cg12101586 | 15 | CYP1A1 | 0.351 | 2.74E-08^a^ | 0.283 | 2.31E-04 | 0.479 | 3.43E-05 |
| cg22549041 | 15 | CYP1A1 | 0.314 | 7.67E-10^a^ | 0.274 | 1.10E-05 | 0.451 | 1.68E-06 |
| cg11924019 | 15 | CYP1A1 | 0.440 | 7.49E-11^a^ | 0.337 | 3.68E-05 | 0.646 | 3.99E-07 |
| cg18092474 | 15 | CYP1A1 | 0.474 | 6.28E-12^a^ | 0.417 | 4.85E-07^a^ | 0.581 | 2.09E-06 |
| cg10799388 | 16 | JMJD8 | -0.272 | 2.06E-05 | -0.394 | 3.30E-07^a^ | -0.142 | 0.281 |
| cg17521795 | 16 | ZNF747 | -0.384 | 2.20E-07^a^ | -0.427 | 3.81E-06 | -0.143 | 0.291 |
| cg03776506 | 16 | ZNF688 | -0.232 | 9.65E-06 | -0.323 | 2.20E-07^a^ | -0.062 | 0.553 |
| cg00496272 | 16 | CENPT;THAP11 | -0.230 | 5.07E-05 | -0.347 | 7.02E-07^a^ | -0.090 | 0.412 |
| cg22640868 | 17 | TNFAIP1;IFT20 | -0.165 | 1.71E-05 | -0.219 | 1.71E-06^a^ | 0.014 | 0.854 |
| cg03513978 | 18 | CDH2 | -0.391 | 4.06E-07^a^ | -0.332 | 3.57E-04 | -0.278 | 0.063 |
| cg22332722 | 18 | CDH2 | -0.394 | 1.22E-06^a^ | -0.316 | 9.96E-04 | -0.301 | 0.065 |
| cg18625289 | 19 | MBD3 | -0.281 | 6.04E-05 | -0.437 | 5.62E-07^a^ | -0.201 | 0.123 |
| cg08158952 | 19 | ZNF835 | -0.365 | 1.66E-07^a^ | -0.324 | 1.06E-04 | -0.408 | 0.002 |
| cg07339236 | 20 | ATP9A | -0.425 | 9.16E-10^a^ | -0.475 | 2.00E-08^a^ | -0.425 | 6.32E-04 |
| cg00500729 | 21 | POFUT2;LOC642852 | -0.304 | 4.43E-05 | -0.439 | 1.07E-06^a^ | -0.115 | 0.416 |
| cg08625065 | 22 | UFD1L | -0.368 | 2.70E-07^a^ | -0.375 | 1.08E-05 | -0.265 | 0.053 |

^a^ FDR < 0.05

^*^ By testing smoking×race interactions on epigenome-wide DNAm, we found a significant interaction with CpG site cg14650464 in the *FANCC* gene.

^$^All models adjusted for maternal age, education level, race/ethnicity, parity, pre-pregnancy BMI, alcohol consumption, gestational age, pregestational/gestational diabetes, child sex, cord blood cell compositions (CD8, CD4, NK, B cell, monocytes, granulocytes, nucleated red blood cells).

^+^All models adjusted for maternal age, education level, parity, pre-pregnancy BMI, alcohol consumption, gestational age, pregestational/gestational diabetes, child sex, cord blood cell compositions (CD8, CD4, NK, B cell, monocytes, granulocytes, nucleated red blood cells).

**Supplemental Table 3.** Methylation mediation effect* of each of the 38 single CpG sites on the maternal smoking-newborn birthweight association in 954 mother-newborn pairs from the Boston Birth Cohort.

| **Gene** | **CpG** | **Mediated Effect**  **(VanderWeele-Vansteelandt Approach)** | | | |
| --- | --- | --- | --- | --- | --- |
|  |  | **Beta** | **SE** | **P** | **% Mediated** |
| **GFI1** | cg09662411 | -59.6 | 20.7 | 0.004 | 38.5 |
|  | cg06338710 | -47.7 | 20.8 | 0.022 | 30.0 |
|  | cg18146737 | -60.3 | 23.4 | 0.010 | 38.8 |
|  | cg18316974 | -61.0 | 25.8 | 0.018 | 38.5 |
|  | cg04535902 | -63.9 | 21.5 | 0.003 | 40.4 |
|  | cg09935388 | -58.0 | 23.0 | 0.012 | 36.9 |
|  | cg14179389 | -35.0 | 16.7 | 0.036 | 22.2 |
|  | GFI1 gene score | -70.1 | 24.0 | 0.003 | 44.6 |
| **AHRR** | cg05575921 | -61.7 | 28.3 | 0.029 | 38.5 |
|  | cg21161138 | -12.0 | 25.0 | 0.631 | 7.7 |
| **CYP1A1** | cg05549655 | -49.1 | 15.4 | 0.001 | 30.7 |
|  | cg17852385 | -28.9 | 12.4 | 0.020 | 18.0 |
|  | cg13570656 | -30.9 | 12.5 | 0.014 | 19.3 |
|  | cg12101586 | -39.1 | 14.8 | 0.008 | 24.5 |
|  | cg22549041 | -32.5 | 13.9 | 0.019 | 20.5 |
|  | cg11924019 | -32.4 | 12.9 | 0.012 | 20.4 |
|  | cg18092474 | -57.5 | 16.9 | 0.001 | 35.8 |
|  | CYP1A1 gene score | -47.9 | 15.5 | 0.002 | 29.8 |
| **DHRS3** | cg03546360 | -8.1 | 19.2 | 0.672 | 5.1 |
| **PRPF38B** | cg18011080 | -21.9 | 14.4 | 0.128 | 14.1 |
| **MEIS1** | cg08791563 | -9.3 | 15.2 | 0.540 | 6.0 |
| **ST3GAL5** | cg26537885 | -7.6 | 14.1 | 0.588 | 4.9 |
| **GARS** | cg16875104 | -26.6 | 16.1 | 0.100 | 17.2 |
| **CNTNAP2** | cg25949550 | 0.9 | 12.0 | 0.941 | 0.5 |
| **FBXO16** | cg12474312 | 4.4 | 13.3 | 0.742 | 2.8 |
| **POLR1E** | cg14035521 | -22.3 | 15.0 | 0.137 | 14.4 |
| **GCNT3** | cg25311764 | -21.9 | 14.1 | 0.121 | 14.2 |
| **ZNF747** | cg17521795 | -24.8 | 15.5 | 0.109 | 15.8 |
| **CDH2** | cg03513978 | -14.7 | 15.3 | 0.337 | 9.5 |
|  | cg22332722 | -12.6 | 11.8 | 0.289 | 8.1 |
| **ZNF835** | cg08158952 | -16.2 | 12.6 | 0.199 | 10.2 |
| **ATP9A** | cg07339236 | -16.2 | 13.6 | 0.233 | 10.3 |
| **UFD1L** | cg08625065 | -10.6 | 11.1 | 0.341 | 6.7 |
| **Intergenic CpGs** | cg06415891 | -9.5 | 15.2 | 0.532 | 6.0 |
|  | cg21010202 | -10.7 | 13.9 | 0.442 | 6.8 |
|  | cg04763519 | -11.2 | 15.1 | 0.459 | 7.2 |
|  | cg04565473 | -12.1 | 15.0 | 0.421 | 7.8 |
|  | cg04598670 | -12.4 | 12.5 | 0.322 | 8.0 |
|  | cg27447574 | -1.0 | 7.5 | 0.892 | 0.6 |
|  | cg24061125 | -4.0 | 5.7 | 0.486 | 2.5 |

*All models adjusted for maternal age, education level, race/ethnicity, parity, pre-pregnancy BMI, alcohol consumption, gestational age, pregestational/gestational diabetes, child sex, cord blood cell compositions (CD8, CD4, NK, B cell, monocytes, granulocytes, nucleated red blood cells).

**Supplemental Table 4.** Single CpG, single gene score, and combined multiple gene mediation analysis on the maternal smoking—newborn birthweight association, **among 504 male newborns** in the Boston Birth Cohort

| **Gene** | **CpG** | **Mediated Effect**  **(VanderWeele-Vansteelandt Approach)** | | | |
| --- | --- | --- | --- | --- | --- |
|  |  | **Beta** | **SE** | **P** | **% Mediated** |
| **GFI1** | cg09662411 | -64.4 | 32.9 | 0.050 | 46.3 |
|  | cg06338710 | -42.8 | 33.0 | 0.195 | 30.5 |
|  | cg18146737 | -70.2 | 39.1 | 0.072 | 50.9 |
|  | cg18316974 | -67.1 | 42.3 | 0.113 | 48.0 |
|  | cg04535902 | -67.3 | 29.4 | 0.022 | 48.3 |
|  | cg09935388 | -67.3 | 40.8 | 0.099 | 47.8 |
|  | cg14179389 | -52.9 | 33.8 | 0.118 | 38.1 |
|  | **GFI1 gene score** | -78.1 | 38.8 | 0.044 | **56.4** |
| **AHRR** | cg05575921 | -61.3 | 35.9 | 0.088 | 42.7 |
|  | cg21161138 | -9.7 | 34.5 | 0.780 | 6.9 |
| **CYP1A1** | cg05549655 | -54.1 | 22.3 | 0.015 | 36.8 |
|  | cg17852385 | -38.5 | 20.3 | 0.058 | 26.4 |
|  | cg13570656 | -36.6 | 19.5 | 0.061 | 25.1 |
|  | cg12101586 | -40.0 | 21.6 | 0.064 | 27.6 |
|  | cg22549041 | -37.5 | 21.5 | 0.082 | 26.3 |
|  | cg11924019 | -40.8 | 20.4 | 0.046 | 30.0 |
|  | cg18092474 | -73.1 | 24.4 | 0.003 | 50.7 |
|  | **CYP1A1 gene score** | -57.2 | 24.3 | 0.019 | **39.3** |

| **Parameters** | **Mediation effect by Structural Equation Model**  **(Simultaneous consideration of the 3 genes)** | | |
| --- | --- | --- | --- |
|  | **Beta** | **SE** | **P** |
| **Direct effect of maternal smoking** | -28.0 | 55.7 | 0.615 |
| **Indirect effect via *GFI1* Gene Score** | -29.2 | 18.2 | 0.109 |
| **Indirect effect via *AHRR* CpG cg05575921** | -63.9 | 18.3 | <0.001 |
| **Indirect effect via *CYP1A1* Gene Score** | -17.3 | 10.6 | 0.102 |
| **Sum indirect effect (or Mediation)** | -110.5 | 25.4 | <0.001 |
| **Total effect of maternal smoking** | -138.5 | 55.0 | 0.012 |
|  |  |  |  |
| **Percent Mediated** | **79.8%** |  |  |

*All models adjusted for maternal age, education level, race/ethnicity, parity, pre-pregnancy BMI, alcohol consumption, gestational age, pregestational/gestational diabetes, cord blood cell compositions (CD8, CD4, NK, B cell, monocytes, granulocytes, nucleated red blood cells).

**Supplemental Table 5.** Single CpG, single gene score, and combined multiple gene mediation analysis on the maternal smoking—newborn birthweight association, **among 450 female newborns** in the Boston Birth Cohort

| **Gene** | **CpG** | **Mediated Effect**  **(VanderWeele-Vansteelandt Approach)** | | | |
| --- | --- | --- | --- | --- | --- |
|  |  | **Beta** | **SE** | **P** | **% Mediated** |
| **GFI1** | cg09662411 | -55.2 | 30.0 | 0.066 | 31.7 |
|  | cg06338710 | -56.4 | 29.7 | 0.057 | 31.0 |
|  | cg18146737 | -60.7 | 30.9 | 0.049 | 34.0 |
|  | cg18316974 | -69.8 | 32.7 | 0.033 | 38.3 |
|  | cg04535902 | -72.4 | 32.3 | 0.025 | 40.5 |
|  | cg09935388 | -51.1 | 25.8 | 0.048 | 29.3 |
|  | cg14179389 | -29.9 | 18.8 | 0.113 | 16.7 |
|  | *GFI1* gene score | -71.7 | 31.2 | 0.022 | 40.2 |
| **AHRR** | cg05575921 | -88.7 | 50.0 | 0.076 | 48.9 |
|  | cg21161138 | -11.8 | 36.0 | 0.742 | 6.7 |
| **CYP1A1** | cg05549655 | -78.7 | 39.3 | 0.045 | 43.0 |
|  | cg17852385 | -31.7 | 29.1 | 0.275 | 17.6 |
|  | cg13570656 | -37.7 | 25.0 | 0.131 | 20.8 |
|  | cg12101586 | -44.3 | 29.2 | 0.129 | 24.7 |
|  | cg22549041 | -35.4 | 26.5 | 0.181 | 19.6 |
|  | cg11924019 | -44.5 | 28.4 | 0.117 | 24.2 |
|  | cg18092474 | -72.8 | 37.9 | 0.055 | 39.8 |
|  | *CYP1A1* gene score | -64.2 | 34.8 | 0.065 | 35.1 |

| **Parameters** | **Mediation effect by Structural Equation Model**  **(Simultaneous consideration of the 3 genes)** | | |
| --- | --- | --- | --- |
|  | **Beta** | **SE** | **P** |
| **Direct effect of maternal smoking** | -85.9 | 71.8 | 0.232 |
| **Indirect effect via *GFI1* Gene Score** | -28.9 | 15.2 | 0.057 |
| **Indirect effect via *AHRR* CpG cg05575921** | -47.1 | 22.9 | 0.039 |
| **Indirect effect via *CYP1A1* Gene Score** | -18.2 | 14.8 | 0.219 |
| **Sum indirect effects (or Mediation)** | -94.2 | 31.1 | 0.002 |
| **Total effect of maternal smoking** | -180.1 | 63.3 | 0.004 |
|  |  |  |  |
| **Percent Mediated** | **52.3** |  |  |

*All models adjusted for maternal age, education level, race/ethnicity, parity, pre-pregnancy BMI, alcohol consumption, gestational age, pregestational/gestational diabetes, cord blood cell compositions (CD8, CD4, NK, B cell, monocytes, granulocytes, nucleated red blood cells).

**Supplemental Table 6.** Single CpG, single gene score, and combined multiple gene mediation analysis on the maternal smoking—newborn birthweight association, **among 679 Black newborns** in the Boston Birth Cohort

| **Gene** | **CpG** | **Mediated Effect**  **(VanderWeele-Vansteelandt Approach)** | | | |
| --- | --- | --- | --- | --- | --- |
|  |  | **Beta** | **SE** | **P** | **% Mediated** |
| **GFI1** | cg09662411 | -61.1 | 24.1 | 0.011 | 43.9 |
|  | cg06338710 | -58.1 | 24.4 | 0.017 | 39.4 |
|  | cg18146737 | -74.8 | 29.7 | 0.012 | 52.2 |
|  | cg18316974 | -72.1 | 30.6 | 0.018 | 48.3 |
|  | cg04535902 | -82.9 | 26.0 | 0.001 | 56.9 |
|  | cg09935388 | -67.1 | 26.11 | 0.010 | 45.1 |
|  | cg14179389 | -32.8 | 18.4 | 0.075 | 22.1 |
|  | **GFI1 gene score** | -85.0 | 27.9 | 0.002 | **58.6** |
| **AHRR** | cg05575921 | -91.1 | 39.7 | 0.022 | 58.5 |
|  | cg21161138 | -8.1 | 37.1 | 0.827 | 5.4 |
| **CYP1A1** | cg05549655 | -46.0 | 19.3 | 0.017 | 30.1 |
|  | cg17852385 | -38.9 | 17.4 | 0.026 | 25.1 |
|  | cg13570656 | -53.9 | 21.3 | 0.011 | 33.6 |
|  | cg12101586 | -49.9 | 22.2 | 0.025 | 32.3 |
|  | cg22549041 | -37.6 | 19.9 | 0.059 | 25.0 |
|  | cg11924019 | -30.9 | 16.3 | 0.058 | 20.6 |
|  | cg18092474 | -70.7 | 24.4 | 0.004 | 47.0 |
|  | **CYP1A1 gene score** | -56.2 | 22.1 | 0.011 | **36.4** |

| **Parameters** | **Mediation effect by Structural Equation Model**  **(Simultaneous consideration of the 3 genes)** | | |
| --- | --- | --- | --- |
|  | **Beta** | **SE** | **P** |
| **Direct effect of maternal smoking** | -30.0 | 51.4 | 0.559 |
| **Indirect effect via GFI1 Gene Score** | -40.2 | 13.1 | 0.002 |
| **Indirect effect via AHRR CpG cg05575921** | -54.3 | 16.0 | 0.001 |
| **Indirect effect via CYP1A1 Gene Score** | -22.6 | 10.9 | 0.037 |
| **Sum indirect effects (or Mediation)** | -117.1 | 22.6 | <0.001 |
| **Total effect of maternal smoking** | -147.1 | 49.7 | 0.003 |
|  |  |  |  |
| **Percent Mediated** | **79.6%** |  |  |

*All models adjusted for maternal age, education level, parity, pre-pregnancy BMI, alcohol consumption, gestational age, pregestational/gestational diabetes, child sex, cord blood cell compositions (CD8, CD4, NK, B cell, monocytes, granulocytes, nucleated red blood cells).

**Supplemental Table 7.** Single CpG, single gene score, and combined multiple gene mediation analysis on the maternal smoking—newborn birthweight association, **among 275 non-Black newborns** in the Boston Birth Cohort.

| **Gene** | **CpG** | **Mediated Effect**  **(VanderWeele-Vansteelandt Approach)** | | | |
| --- | --- | --- | --- | --- | --- |
|  |  | **Beta** | **SE** | **P** | **% Mediated** |
| **GFI1** | cg09662411 | -64.5 | 55.4 | 0.243 | 32.7 |
|  | cg06338710 | -18.9 | 52.1 | 0.718 | 9.9 |
|  | cg18146737 | -56.7 | 52.9 | 0.284 | 29.3 |
|  | cg18316974 | -79.6 | 55.8 | 0.154 | 28.4 |
|  | cg04535902 | -59.6 | 45.0 | 0.186 | 30.3 |
|  | cg09935388 | -69.6 | 59.0 | 0.239 | 35.8 |
|  | cg14179389 | -52.7 | 44.0 | 0.231 | 27.5 |
|  | **GFI1 score** | -71.1 | 56.7 | 0.210 | **36.2** |
| **AHRR** | cg05575921 | -19.2 | 45.8 | 0.675 | 10.4 |
|  | cg21161138 | -10.2 | 25.2 | 0.684 | 5.5 |
| **CYP1A1** | cg05549655 | -69.7 | 36.3 | 0.054 | 36.3 |
|  | cg17852385 | -20.0 | 23.8 | 0.400 | 10.5 |
|  | cg13570656 | -3.1 | 15.3 | 0.841 | 1.6 |
|  | cg12101586 | -19.1 | 22.6 | 0.398 | 10.2 |
|  | cg22549041 | -22.8 | 22.4 | 0.309 | 12.2 |
|  | cg11924019 | -49.8 | 30.5 | 0.103 | 26.0 |
|  | cg18092474 | -33.9 | 31.9 | 0.287 | 17.8 |
|  | CYP1A1 score | -37.2 | 29.6 | 0.210 | 19.6 |

| **Parameters** | **Mediation effect by Structural Equation Model**  **(Simultaneous consideration of the 3 genes)** | | |
| --- | --- | --- | --- |
|  | **Beta** | **SE** | **P** |
| **Direct effect of maternal smoking** | -101.7 | 82.1 | 0.215 |
| **Indirect effect via GFI1 Gene Score** | -8.2 | 25.4 | 0.747 |
| **Indirect effect via AHRR CpG cg05575921** | -66.6 | 28.1 | 0.018 |
| **Indirect effect via CYP1A1 Gene Score** | -12.3 | 19.1 | 0.521 |
| **Sum indirect effects (or Mediation)** | -87.1 | 36.8 | 0.018 |
| **Total effect of maternal smoking** | -188.8 | 75.1 | 0.012 |
|  |  |  |  |
| **Percent Mediated** | **46.1%** |  |  |

*All models adjusted for maternal age, education level, parity, pre-pregnancy BMI, alcohol consumption, gestational age, pregestational/gestational diabetes, child sex, cord blood cell compositions (CD8, CD4, NK, B cell, monocytes, granulocytes, nucleated red blood cells).

**Supplemental Table 8**. Characteristics of mother-newborn pairs included versus excluded from this current study.

|  | Included (n=954) | Excluded (n=7,555) | P |
| --- | --- | --- | --- |
| **Continuous Variables (mean (SD))** |  |  |  |
| Maternal age (years) | 28.34 (6.55) | 28.15 (6.45) | 0.389 |
| Maternal pre-pregnancy BMI | 26.98 (6.48) | 25.85 (6.22) | <0.001 |
| Gestational age (weeks) | 38.59 (2.49) | 37.85 (3.26) | <0.001 |
| Birthweight (grams) | 3124 (667) | 2935 (779) | <0.001 |
| **Categorical Variables, n(%)** |  |  |  |
| Parity (>= 1 live birth) | 529 (55.5) | 4317 (57.1) | 0.338 |
| Maternal alcohol consumption (current drinking) | 79 (8.3) | 657 (8.7) | 0.712 |
| Maternal smoking status (current smoking) | 165 (17.3) | 1471 (19.5) | 0.118 |
| Maternal education level (>high school) | 319 (33.4) | 2607 (34.5) | 0.536 |
| Maternal Diabetes |  |  | 0.119 |
| No | 841 (88.2) | 6785 (89.8) |  |
| Gestational Diabetes | 67 (7.0) | 509 (6.7) |  |
| Pregestational Diabetes | 46 (4.8) | 261 (3.5) |  |
| Maternal race |  |  | <0.001 |
| Black | 679 (71.2) | 3352 (44.4) |  |
| Non-Black | 275 (28.8) | 4203 (55.6) |  |
| Child sex (male) | 504 (52.8) | 3733 (49.4) | 0.05 |

**Supplemental Figure 1.** Illustration of the relationship between maternal smoking categories (0=never, 1= quitter, 2=current) and *GFI1* gene score (Panel A), *AHRR* cg05575921 (Panel B), and *CYP1A1* gene score (Panel C); and between smoking categories (0=never, 1= quitter, 2=current) and birthweight (Panel D), in 954 mother-newborn pairs from the Boston Birth Cohort.

| (A)  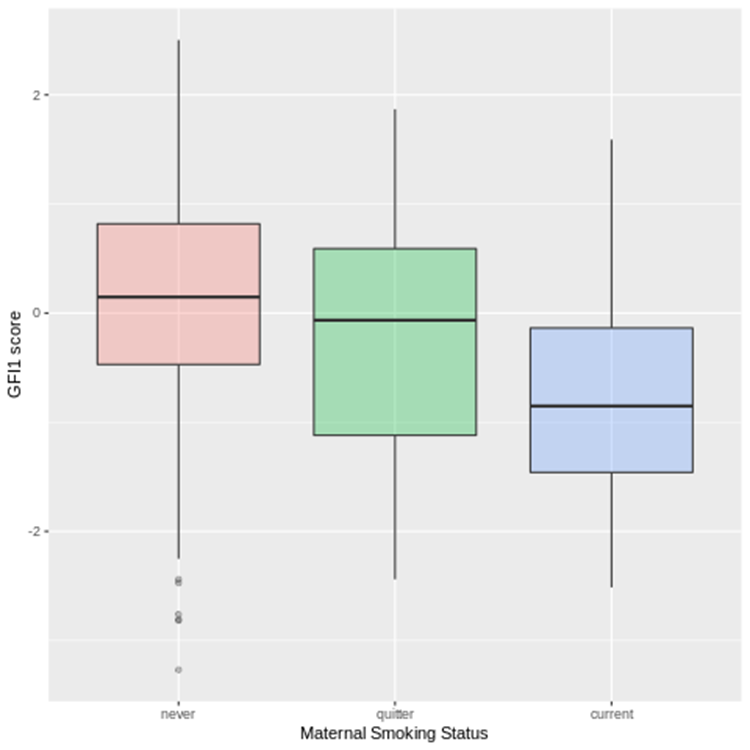 | (B) 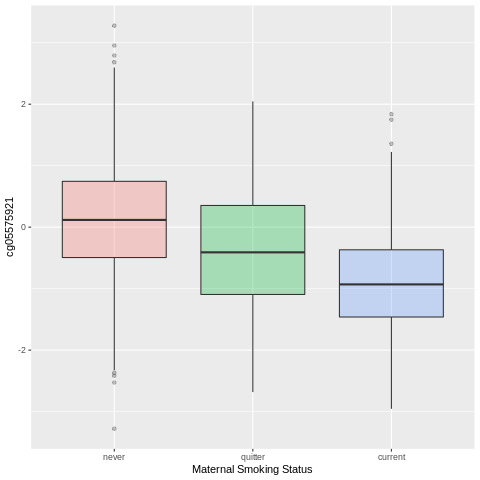 |
| --- | --- |
| (C) 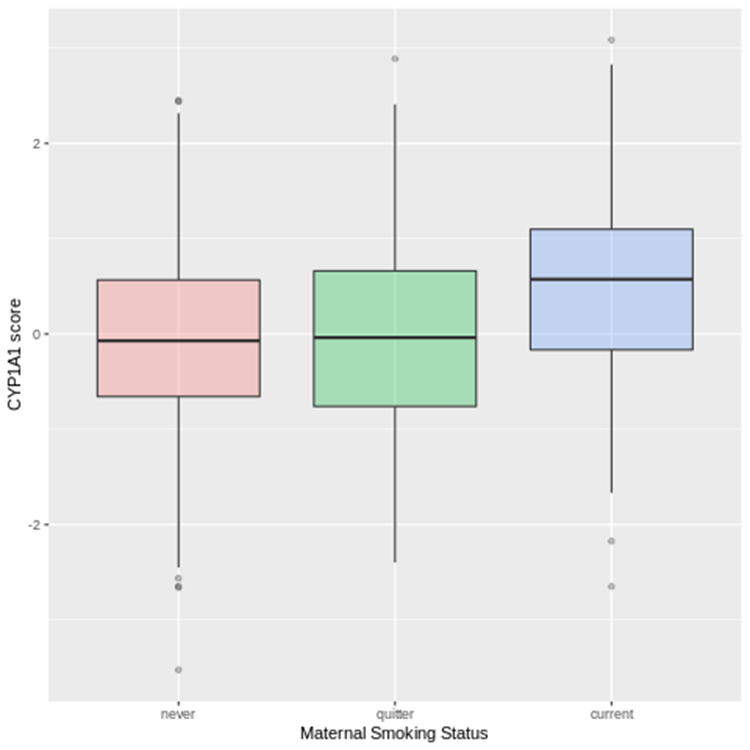 | (D)  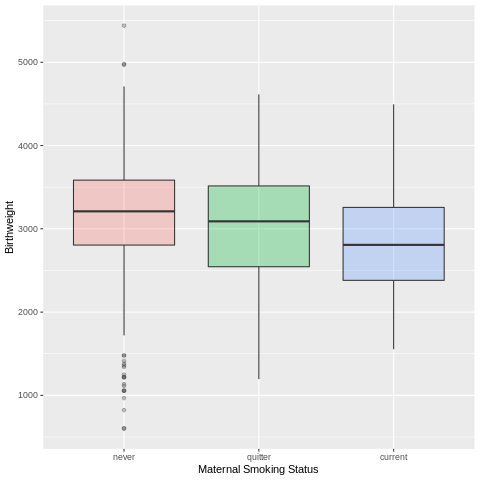 |

**Supplemental Figure 2**. Manhattan and Q-Q plots for the EWA analyses in cord blood in associations with maternal smoking, in Black (left panel) and non-Black subset (right panel). The analyses adjusted for maternal age, education level, parity, pre-pregnancy BMI, alcohol consumption, gestational age, pregestational/gestational diabetes, child sex, cord blood cell compositions (CD8, CD4, NK, B cell, monocytes, granulocytes, nucleated red blood cells).

| Blacks | non-Blacks |
| --- | --- |
| 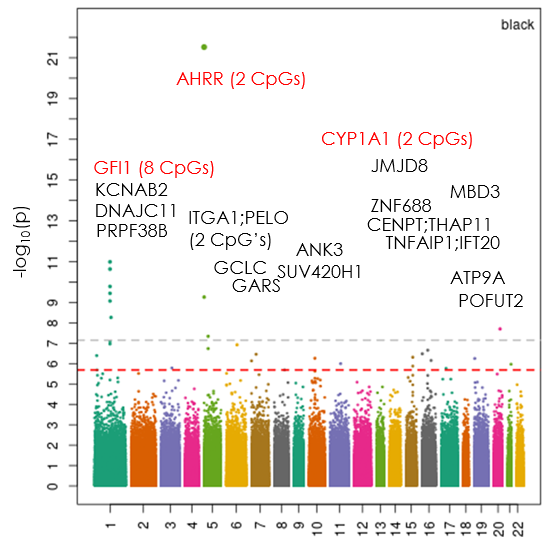 | 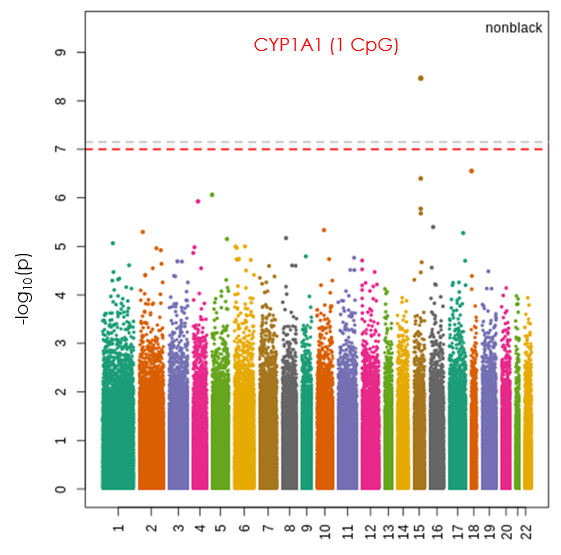 |
| 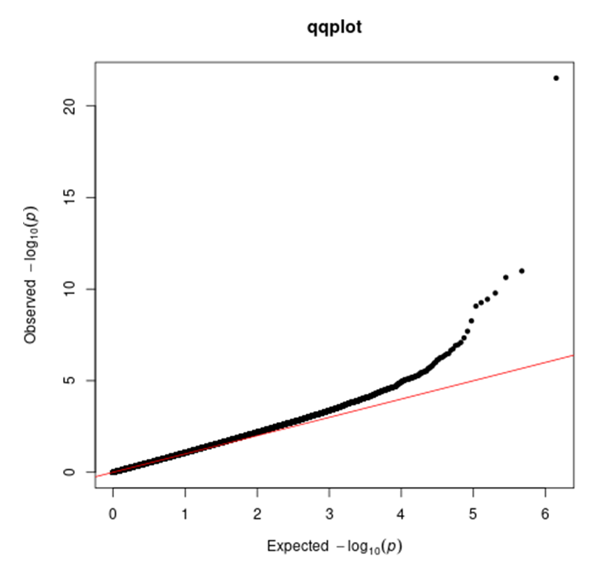 | 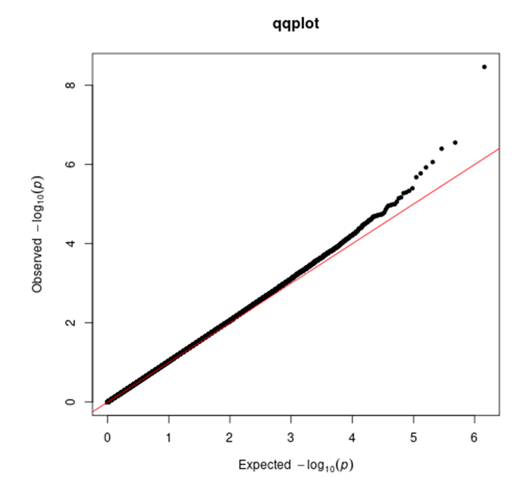 |

**Supplemental Figure 3**. An illustration of our sequential analytical methods to dissect the interplay of maternal smoking (X), cord blood DNAm (M: mediator), and newborn birthweight (Y), where C’ represents direct effect of X on Y.

Single Mediator

VanderWeele & Vansteelandt Approach

Multiple mediators

Structural Equation Models


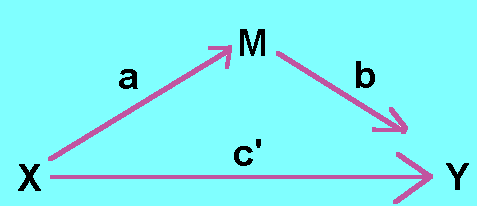


EWAS to identify significant CpGs associated with maternal smoking

Association of fetal DNAm with Birthweight

**Supplemental Figure 4. Distribution of Cotinine/Hydroxy-cotinine by Smoking Status**

| Panel A: Cord Plasma Cotinine / Hydroxy-cotinine by Maternal Smoking Status | |
| --- | --- |
| 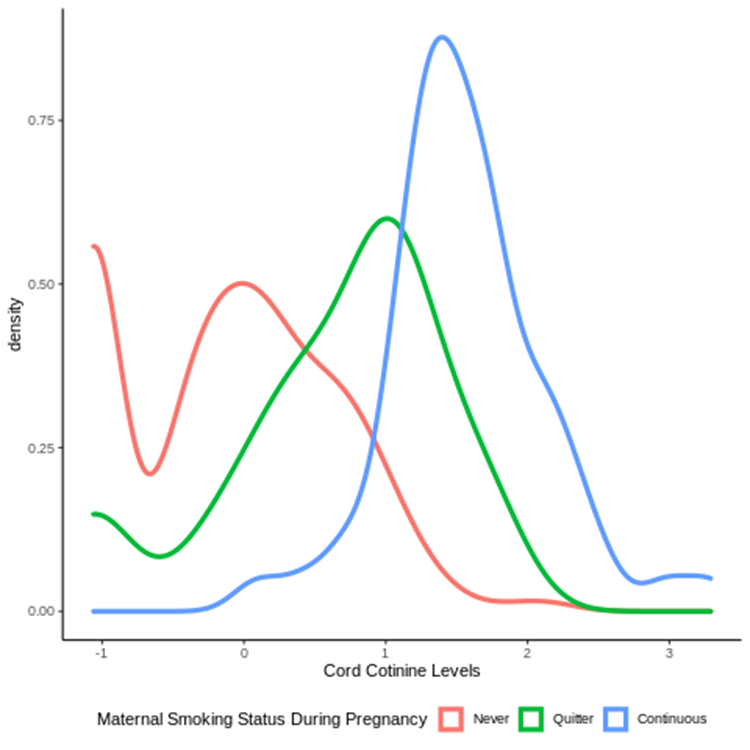 | 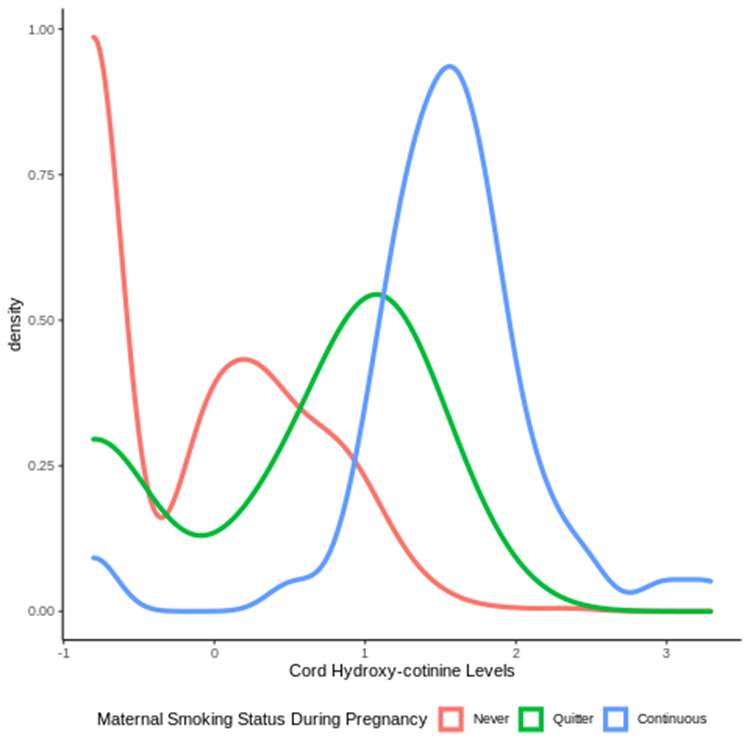 |
|  |  |
| 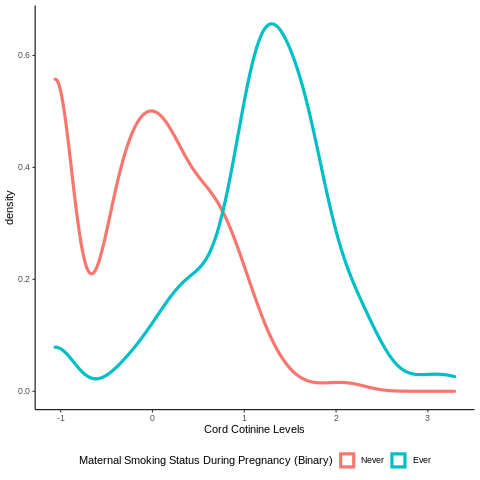 | 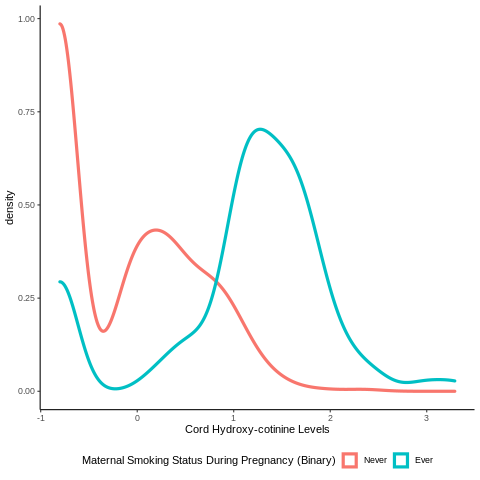 |

| Panel B: Maternal Plasma Cotinine / Hydroxy-cotinine by Maternal Smoking Status | |
| --- | --- |
| 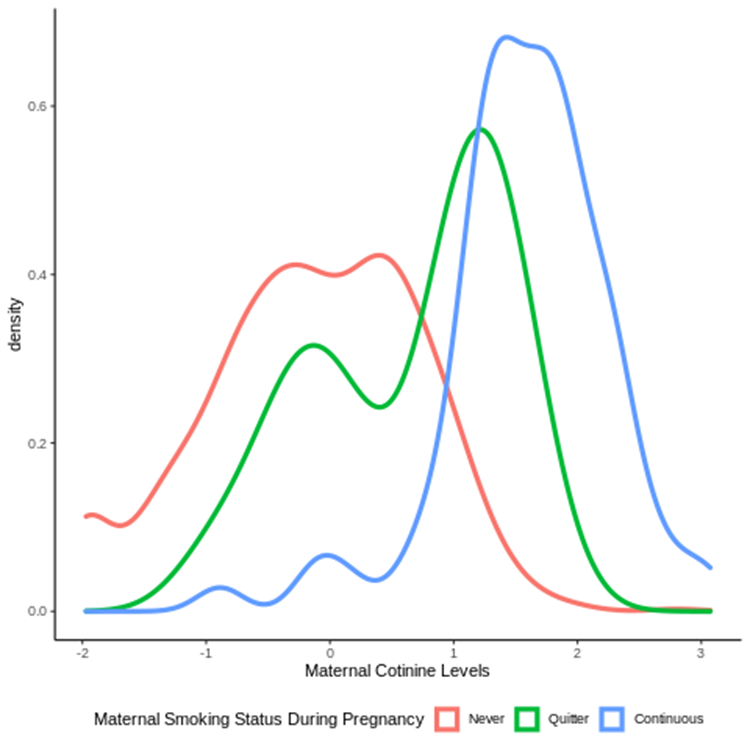 | 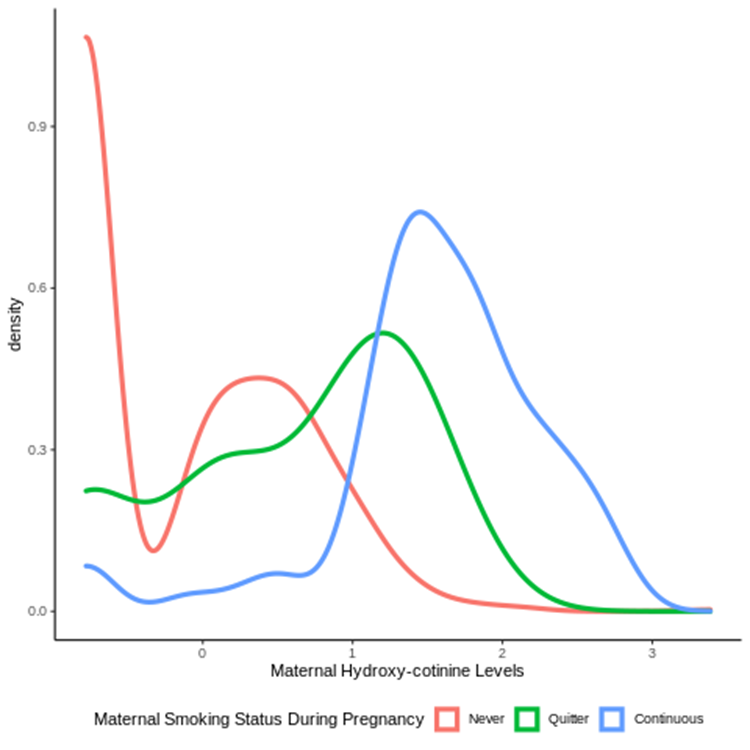 |
|  |  |
| 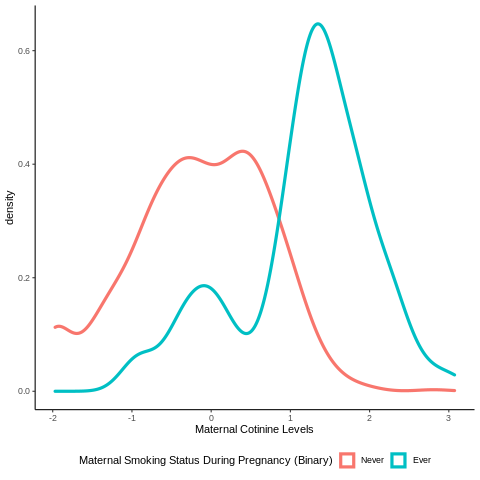 | 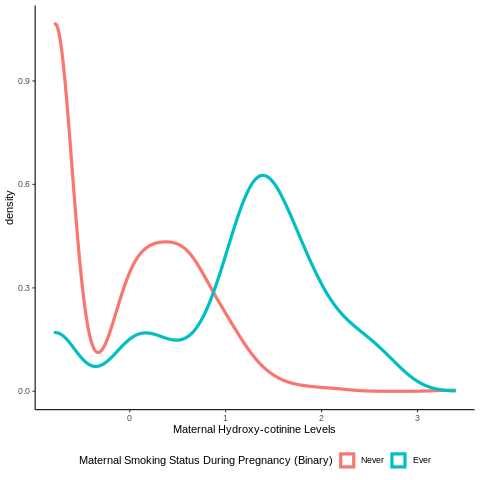 |

**Supplemental Figure 5.** Flowchart of Study Participants

n=8,509
Mother-newborn pairs enrolled in the Boston Birth Cohort

n=7,555
Excluded because of lack of cord DNA methylation data and other reasons*

n=954
Children with cord DNA methylation data

*****The excluded samples include children who did not continue medical care at the Boston Medical Center, who did not have sufficient cord blood DNA samples, who did not have electronic medical record data, or who did not consent for postnatal follow-up at the time of sample selection for DNA methylation assay.
